# Metabolic and Mitochondrial Dysregulations in Diabetic Cardiac Complications

**DOI:** 10.3390/ijms26073016

**Published:** 2025-03-26

**Authors:** Asim J. Tashkandi, Abigail Gorman, Eva McGoldrick Mathers, Garrett Carney, Andrew Yacoub, Wiwit Ananda Wahyu Setyaningsih, Refik Kuburas, Andriana Margariti

**Affiliations:** Wellcome Wolfson Institute of Experimental Medicine, Queens University Belfast, Belfast BT9 7BL, Northern Ireland, UK; atashkandi01@qub.ac.uk (A.J.T.); agorman14@qub.ac.uk (A.G.); emcgoldrickmathers01@qub.ac.uk (E.M.M.); gcarney03@qub.ac.uk (G.C.); ayacoub01@qub.ac.uk (A.Y.); wsetyaningsih01@qub.ac.uk (W.A.W.S.)

**Keywords:** diabetes, cardiomyocytes, IPSCs, organoids, hyperglycaemia, lipotoxicity, mitochondrial dysfunction, AMPK, RNA binding proteins, extracellular vesicles

## Abstract

The growing prevalence of diabetes highlights the urgent need to study diabetic cardiovascular complications, specifically diabetic cardiomyopathy, which is a diabetes-induced myocardial dysfunction independent of hypertension or coronary artery disease. This review examines the role of mitochondrial dysfunction in promoting diabetic cardiac dysfunction and highlights metabolic mechanisms such as hyperglycaemia-induced oxidative stress. Chronic hyperglycaemia and insulin resistance can activate harmful pathways, including advanced glycation end-products (AGEs), protein kinase C (PKC) and hexosamine signalling, uncontrolled reactive oxygen species (ROS) production and mishandling of Ca^2+^ transient. These processes lead to cardiomyocyte apoptosis, fibrosis and contractile dysfunction. Moreover, endoplasmic reticulum (ER) stress and dysregulated RNA-binding proteins (RBPs) and extracellular vesicles (EVs) contribute to tissue damage, which drives cardiac function towards heart failure (HF). Advanced patient-derived induced pluripotent stem cell (iPSC) cardiac organoids (iPS-COs) are transformative tools for modelling diabetic cardiomyopathy and capturing human disease’s genetic, epigenetic and metabolic hallmarks. iPS-COs may facilitate the precise examination of molecular pathways and therapeutic interventions. Future research directions encourage the integration of advanced models with mechanistic techniques to promote novel therapeutic strategies.

## 1. Introduction

As diabetes rates continue to rise and the population ages, developing new models for identifying therapeutic targets and conducting preclinical testing is vital. Diabetes is a multifactorial and multisystemic disease that affects the entire body. Its repercussions on the cardiovascular system are especially significant, highlighted by the increased rates of heart disease and heart failure among diabetic patients.

In this review, we will examine the underlying mechanisms of diabetic cardiac complications, emphasising several molecular pathways and the use of patient-induced pluripotent stem cell (iPSC)-derived organoids as a human diabetic model in advancing our understanding and elucidating these pathological pathways and to explore novel therapeutic targets.

### 1.1. Epidemiology of Diabetes

Diabetes mellitus is a substantial global health burden estimated to affect half a billion people by 2030 [1]. The two types of diabetes cause alterations in the systemic metabolic milieu, leading to injury in multiple organs, including the heart. Type I diabetes mellitus results from the destruction of pancreatic β-cells induced via genetic susceptibility and environmental factors [2]. The more common type, type II diabetes mellitus (T2DM), results from insulin resistance due to functional defects of β-cells and impaired insulin sensitivity and cell uptake and is highly connected to a diet rich in sugars and fat and a sedentary lifestyle.

Diabetes-associated mortality rates continue to rise, whereas mortality rates of cancer have decreased within the past 20 years. Moreover, diabetes is known to directly contribute to or exacerbate conditions such as cardiovascular disease, including stroke, kidney failure, renal failure and blood vessel and nerve damage. Diabetes was the direct cause of 1.6 million deaths and 47% of deaths within individuals over 70 years [3,4].

The treatment of diagnosed diabetic patients with complications in the UK was estimated to be ~£14 billion, constituting 10% of the NHS (National Health Service) budget in 2010 [5]. Similarly, the cost reached $245 billion in the United States in 2012, with statistics rising [6]. These financial statistics highlight the economic impact and emphasise the necessity of developing effective treatment methods and preventative measures for diabetic complications. The ever-increasing prevalence of diabetes and cardiovascular complications highly contributes to diabetes-related morbidity and mortality. It is essential to examine how diabetes-induced metabolic changes directly damage cardiac tissue and understand their complications.

### 1.2. Diabetes-Induced Cardiac Damage

Cardiac toxicity arises from electrophysiological dysfunction or harm to cardiac muscle, leading to myocardial infarction due to insufficient oxygenated blood supply, which results in the death of cardiomyocytes and a decline in myocardial function. Compared to non-diabetic patients, patients with both heart failure (HF) and diabetes are reported to have a poorer prognosis and quality of life and experience worse clinical outcomes following an incidence of myocardial infarction [4,7]. The prevalence of systolic dysfunction was significantly higher in diabetic patients by 157% compared to non-diabetic individuals [8]. Additionally, patients with HF often exhibit insulin resistance, which can trigger or worsen existing diabetes, emphasising that T2DM and HF mutually aggravate one another [9].

Cardiac toxicity induced by T2DM can lead to ischemic stress, a process known to cause metabolic changes. This results in increased production of reactive oxygen species (ROS), Ca^2+^ overload and the depletion of adenosine triphosphate (ATP) availability. Ischemic injury targets the mitochondria for cell death, defecting the electron transport chain and oxidative phosphorylation, and further contributes to the deprivation of energy availability within the myocardium [10,11].

During an ischemic event, altered cytosolic Ca^2+^ regulation within mitochondria induces structural fragility, and excessive contractile activation results in necrotic cell death via mitochondrial permeability transition pore (mPTP) opening. In response to ATP depletion and oxidative stress, the opening of the mPTP channel mediates cardiac myocyte death via the uncoupling of oxidative phosphorylation and swelling of the mitochondria [12]. Following cardiac myocyte and endothelial cell injury, the generation of ROS is known to exacerbate membrane damage, Ca^2+^ loading and the release of inflammatory mediators, all of which contribute to apoptotic cell death in the myocardium.

The cascading events of apoptosis fragment dead cells into membrane-bound apoptotic bodies for phagocytosis via macrophages and surrounding cells, contributing to the loss of the major source of ATP production for energy metabolism. The resulting event initiates anaerobic glycolysis for ATP production, accumulating hydrogen ions and lactate. This toxic accumulation contributes to intracellular acidosis and glycolysis inhibition by promoting mitochondrial fatty acid metabolism [13]. Additionally, mitochondrial dysfunction directly impairs endothelial function, which is critical in maintaining proper coronary blood flow. Endothelial cell dysfunction in diabetes leads to reduced nitric oxide production and endothelial nitric oxide synthase (eNOS) activity, further compounding the cardiovascular risks associated with diabetes [14,15].

Beyond the structural and electrophysiological damage caused by diabetes, here we discuss a distinct form of heart disease known as diabetic cardiomyopathy that develops independently of cardiovascular disease or hypertension in order to understand its unique pathophysiology [16].

### 1.3. Pathophysiology of Diabetic Cardiomyopathy

**Figure 1:**
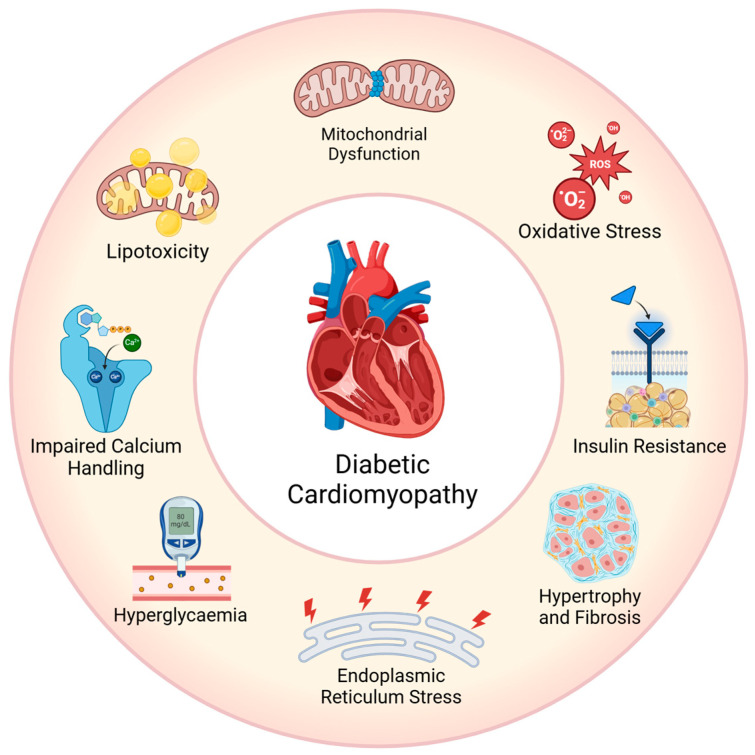
Mechanisms of diabetic cardiomyopathy. Hyperglycaemia and insulin resistance lead to several negative effects on heart cells, including lipotoxicity and Ca^2+^ mishandling, which contribute to mitochondrial dysfunction and endoplasmic reticulum stress. Over time, these interconnected processes exacerbate cardiac dysfunction and the progression of diabetic cardiomyopathy. Created with BioRender.com.

Multiple mechanisms regarding the pathogenesis of diabetic cardiomyopathy were researched, including insulin resistance, lipotoxicity, oxidative stress, inflammation, fibrosis and hypertrophy, as summarised in Figure 1 [17].

A normal heart is metabolically flexible, relying on fatty acid oxidation (FAO) for 70% of its energy, with the remaining energy coming from glucose and other substrates. This flexibility allows the cardiac cells to adjust their energy source during high-demand conditions such as exercise or ischemia, as lipid oxidation requires 12% more oxygen than glucose [18]. However, due to insulin resistance in diabetic conditions, the cardiac cells rely strictly on fatty acids, which causes metabolic rigidity [19,20].

Aggravated FAO and alternative glucose pathways are connected to increased electron transport chain flux, upregulating mitochondrial uncoupling protein and increasing proton leakage. This increases the generation of intracellular ROS that damage cellular proteins and DNA [21,22]. Utilising more fatty acids for energy places greater strain on cardiac cells as they use fuel that requires increased oxygen consumption and mitochondrial uncoupling, resulting in reduced energy efficiency [20]. Excessive circulating lipids in the bloodstream without enough FAO capacity promotes cytosol lipid accumulation, leading to lipotoxicity through the buildup of intermediates such as ceramides and diacylglycerols [23,24].

While insulin-independent pathways such as GLUT1 and sodium/glucose cotransporter 1 (SGLT1) allow a stable influx of glucose into cardiomyocytes, hyperglycaemia results in the accumulation of glucose and glycolysis intermediates in the cytosol [25]. The less glucose undergoes oxidative phosphorylation in the mitochondria, the more hyperglycaemia and hyperlipidaemia promote the activation of molecular pathways such as the production of advanced glycation end-products (AGEs), polyol pathways and hexosamine pathways [26].

The formation of AGEs results in the cross-linking of proteins, which impairs cellular function and increases oxidative stress. The polyol pathway promotes the accumulation of sorbitol and fructose, disrupts the antioxidation balance and exacerbates oxidative stress [27]. In addition, the hexosamine pathway can modify proteins through O-GlcNAcylation and alter cellular signalling [28]. Inflammatory pathways that are primarily driven by protein kinase C (PKC) activation and nuclear factor-kappa B (NFκB) signalling can promote chronic inflammation and further oxidative stress [29]. This cycle of damage accelerates insulin resistance, Ca^2+^ mishandling and mitochondrial dysfunction, all of which contribute to the progression of diabetic cardiomyopathy, as summarised in Figure 1.

Mitochondrial dysfunction in diabetes is characterised by an impaired electron transport chain, elevated ROS production and oxidative damage to mitochondrial DNA (mtDNA) [30,31]. The mitochondrial biogenesis and dynamics are also disrupted, leading to an imbalance in fusion and fission processes. Mitochondria suffer increased susceptibility to mPTP opening, which triggers cell death pathways [32]. These mitochondrial defects can directly impair cardiomyocyte contractility by disrupting Ca^2+^ homeostasis and energy production. In addition, the exacerbated ROS generation from damaged mitochondria contributes to myocardial fibrosis and extracellular matrix remodelling [33].

Several antioxidation pathways, such as AMP-activated protein kinase (AMPK), the Nuclear factor-E2-related factor (NRF2) and the phosphatidylinositol-3-OH kinase/protein kinase B (PI3K/Akt) pathway, handle and counterbalance the increased oxidative stress [34]. The gradual exacerbation of ROS production overwhelms these pathways and even inhibits some, leading to drastic cellular damage. As a result, diabetic individuals are predisposed to structural and functional cardiac symptoms, which manifest as diastolic dysfunction and an increased risk of heart failure.

## 2. Metabolic Disruptions in the Diabetic Heart

### 2.1. Hyperglycaemia and Insulin Resistance

Insulin, a peptide hormone produced by the β-cells of the pancreas, rises in the bloodstream in response to increased blood glucose levels. It promotes glucose uptake in insulin-sensitive tissues through GLUT4 translocation and regulates lipid and protein metabolism. It also has cardioprotective effects that maintain endothelial function, inhibit apoptosis and reduce inflammation. The dysregulation of insulin signalling, such as in insulin resistance and diabetes, can lead to hyperglycaemia and complications like cardiomyopathy.

**Figure 2:**
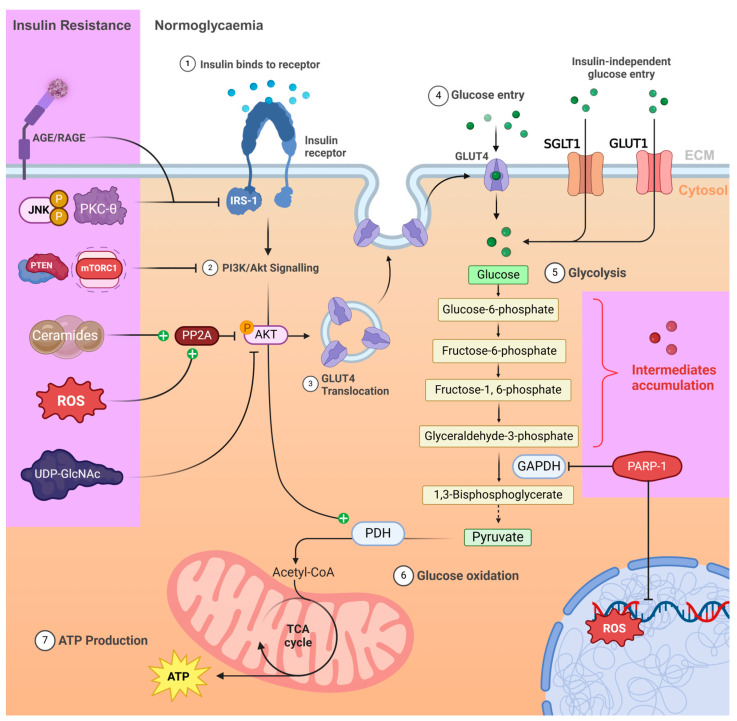
Glucose metabolism under normoglycaemic conditions and insulin resistance. Under the normoglycaemic state, insulin binds to its receptor to initiate the PI3K/Akt signalling cascade, which leads to GLUT4 translocation and glucose uptake (steps 1–4). Glucose undergoes glycolysis (step 5), producing pyruvate, which under pyruvate dehydrogenase is turned into acetyl-CoA to be oxidised in the mitochondria via the TCA cycle to produce ATP (step 6–7). In insulin resistance (shaded in pink), several factors contribute to impaired insulin signalling, such as activation of AGE/RAGE and GlcNAcylation, overstimulation of PKC-θ and PTEN, excess feedback of mTORC1 and overproduction of ceramides and ROS. All these factors result in reduced Akt activation, which prevents GLUT4 translocation and decreases glucose oxidation. If insulin-independent glucose uptake via GLUT1 and SGLT1 persists, it contributes to excess cytosolic glycolytic intermediates. Increased ROS production leads to PARP-1 activation to repair DNA but also targets GAPDH, disrupting glycolysis. Created with BioRender.com.

Hyperglycaemia promotes the activation of four molecular pathways related to glucose-mediated cardiomyopathy: the formation of AGEs, the polyol pathway and the hexosamine pathway, and inflammatory pathways mediated by PKC and NFκB, as summarised in Figure 2 [26].

#### 2.1.1. Physiological Functions of Insulin

Physiologically, insulin regulates glucose uptake and oxidation, as well as glycogen synthesis, growth and the survival of cardiomyocytes [35]. It acts mainly by binding to cell surface insulin receptors, which, through the phosphorylation of its insulin receptor substrates (IRS1/2), activates PI3K that converts phosphatidylinositol 4,5-bisphosphate (PIP2) into phosphatidylinositol 3,4,5-trisphosphate (PIP3), a key regulatory step in the PI3K/Akt signalling pathway [36]. PIP3 recruits Akt to the plasma membrane for full activation, after which it starts the Akt-mediated translocation of glucose transporters type 4 (GLUT4) into the sarcolemma. This transfers glucose into the cytosol to be converted into pyruvate and then into the mitochondria for oxidation to acetyl-coenzyme A (acetyl-CoA) for ATP production through the TCA cycle [37]. Insulin-stimulated mitochondrial Akt was identified as a prerequisite transmitter of the insulin signal that directly stimulates mitochondrial glucose oxidation, independent of increasing glucose uptake or glycolysis, by activating mitochondrial pyruvate dehydrogenase (PDH), the rate-limiting enzyme of glucose oxidation [38].

GLUT4 translocation is also induced by cardiac muscle contraction; thus, the fold increase in glucose uptake in insulin-stimulated contracting hearts is less than the changes observed in skeletal muscle or adipose tissue [39]. Therefore, insulin signalling in cardiomyocytes is not essential for maintaining cardiac metabolism under non-stressful conditions, as insulin-independent pathways, such as GLUT1 and SGLT1, provide a baseline of glucose uptake, even when a lack of insulin or GLUT4 function is compromised. Insulin also promotes the translocation of CD36, the FA transporter, to promote FA intake, although it suppresses mitochondrial FAO by reversing the Randle cycle [40]. Insulin also inhibits the activity of AMPK and increases malonyl CoA, a potent inhibitor of mitochondrial fatty acid uptake [41,42].

Besides the metabolic effect, insulin plays a cardioprotective role by preventing stress-induced apoptosis via the promotion of Akt phosphorylation of the pro-apoptotic B-cell lymphoma 2 (Bcl-2) family member BAD, thereby blocking BAD-induced apoptosis and promoting cell survival [13,43,44,45]. The lack of insulin’s effect on cardiomyocytes, whether due to insulin resistance or the absence of insulin receptors, results in similar impaired metabolic functions in mice studies, strongly impairing glucose utilisation and mitochondrial function and increasing oxidative stress [46].

The insulin signalling interactions are under intricate regulation. Key regulators include Phosphatase and tensin homolog (PTEN) and Protein Phosphatase 2A (PP2A). PTEN is a negative regulator of the PI3K/Akt pathway, dephosphorylating PIP3 (the Akt activator), while PP2A dephosphorylates Akt itself [47,48]. Under physiological conditions, PTEN ensures balanced insulin signalling and regulates energy metabolism through interactions with AMPK and PPARγ. It also prevents the hyperactivation of mTORC1, which could otherwise disrupt metabolic homeostasis [49].

Hyperglycaemia-induced oxidative stress and chronic inflammation can oxidise and activate PTEN and PP2A, resulting in suppressed Akt signalling, impaired glucose oxidation and reduced cardiomyocyte survival [50].

#### 2.1.2. Factors of Insulin Resistance

As diabetes progresses, multiple factors, shown in Figure 2, contribute to the development of insulin resistance by disrupting key signalling pathways and will be discussed later. Oxidative stress, primarily due to ROS and NADPH oxidase and their enhancement by AGE/RAGE activation, can worsen insulin resistance by oxidising Akt and activating PP2A [51]. Inflammatory cytokines, such as PKC-θ and NFκB, also trigger c-Jun N-terminal kinase (JNK) activation, which inhibits IRS-1 [52]. Ceramides, a product of increased lipid storage under hyperglycaemic conditions, oxidise IRS-1 and activate PP2A, both of which impair insulin signalling [53]. The resulting ER stress suppresses both IRS-1 and PI3K activity via the PERK-CHOP pathway [54]. Finally, mTORC1 negative feedback promotes IRS-1 degradation, shutting down insulin signalling [55].

The cytoprotective antiapoptotic feature of the PI3K/Akt pathway mediated by insulin diminishes due to Akt desensitisation [56,57]. High blood glucose levels facilitate glucose entry into the cardiomyocyte through SGLT1, which was found to be upregulated two- to three-fold in T2DM and ischemic hearts [25]. Glucose buildup inside cardiomyocytes enhances glycolysis, a fast anaerobic process, but compared to glucose oxidative phosphorylation in the mitochondria, it is inefficient in ATP yield under high-energy demand.

The action of a major glycolytic enzyme, glyceraldehyde-3-phosphate dehydrogenase (GAPDH), may be disrupted by the PARylation process, which detects and repairs ROS damage to DNA (Figure 2) [58]. This interference results in incomplete glycolysis, and without proper insulin signalling, which mediates glucose oxidative phosphorylation in the mitochondria, glycolytic intermediates accumulate in the cytosol [59]. As more glucose and its derivative accumulate, alternative glucose metabolism pathways are activated, such as the production of AGEs and the hexosamine pathway, contributing to additional cardiac metabolic dysfunction [60].

#### 2.1.3. Advanced Glycation End-Products

**Figure 3:**
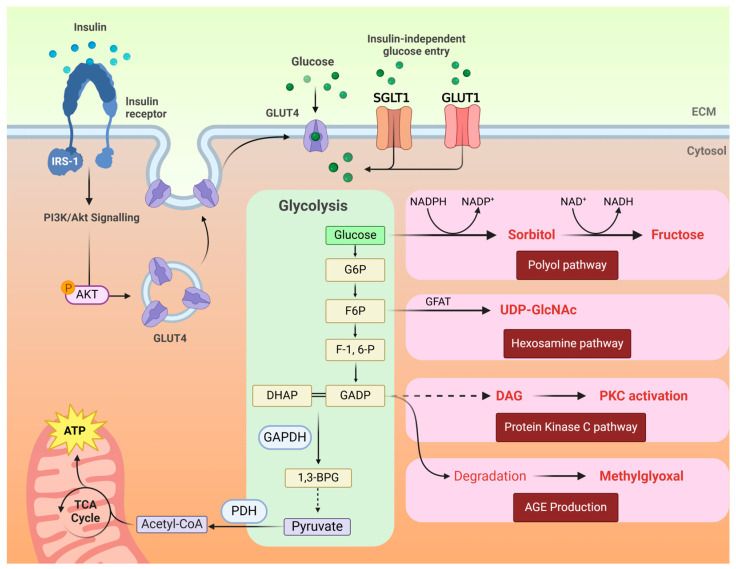
Alternative glucose metabolism pathways in diabetic cardiomyopathy. Chronic hyperglycaemia and insulin resistance set off a series of metabolic disturbances in cardiomyocytes. Excessive glucose uptake through SGLT1 and GLUT1 stimulates the polyol, hexosamine and glycolysis pathways, generating harmful byproducts such as sorbitol, UDP-GlcNAc, methylglyoxal and diacylglycerol. These byproducts lead to ROS formation, which inflicts oxidative stress on mitochondrial glucose oxidation and other cellular pathways. In addition, the diacylglycerol and methylglyoxal buildup can activate inflammatory pathways, including STAT3, NFκB and PKC, worsening cardiac inflammation and fibrosis. Mitochondrial dysfunction also suffers rigid fatty acid oxidation and overload of the electron transport chain. This cycle of oxidative stress, inflammation and metabolic dysfunction leads to deteriorating cardiac tissue integrity and reduced contractility, progressing towards heart failure. Created with BioRender.com.

AGEs provoke glucotoxicity by altering the function of intracellular and extracellular proteins and binding to cell surface AGE receptors (RAGEs) [61]. Methylglyoxal, a highly reactive dicarbonyl compound primarily formed as a byproduct of glycolysis (Figure 3), reacts with free amino groups on proteins, lipids and nucleic acids, forming AGEs that accumulate in tissues over time [62]. Their harmful effect manifests in forming intermolecular crosslinks and irreversibly deactivating structural and functional proteins, rendering them resistant to proteolytic digestion and susceptible to being catalytic sites for ROS production via NADPH oxidases (NOX) [63].

Chronic activation of AGE/ RAGE may influence metabolic memory [64], which maintains persistent RAGE overexpression and sustained NFκB-led inflammation, continuing even after the reversal of hyperglycaemia [65].

AGEs also contribute to contractile dysfunction mainly disrupting both structural and biochemical functions. AGEs induce excessive fibrosis and maladaptive remodelling as they alter internal structural and crosslink extracellular collagen fibres [51,66,67]. AGEs also affect cardiac contractility by crosslinking sarcoplasmic reticulum (SR) proteins, ruining their ability to maintain intracellular Ca^2+^ homeostasis, which is essential for cardiac sarcomere contraction and relaxation [68], impairing myocardial diastole and increasing cardiac stiffness [69].

AGEs also damage the intracellular extrasarcomeric cytoskeleton by promoting abnormal desmin crosslinking and non-enzymatic glycation, disrupting desmin’s flexibility and its striated arrangement [70]. Desmin filaments connect myofibrils and mitochondria at the sarcomere’s Z-disk, and its mutations have been implicated in the onset of dilated cardiomyopathy [71].

Methylglyoxal disrupts mitochondrial respiration and alters specific mitochondrial proteins. Glycating ETC proteins, particularly in Complexes I and III, impairs the standard electron flow, leading to electron leakage and making them more prone to excess superoxide production [72]. This occurs regardless of hyperglycaemia levels, as glycated mitochondria keep generating ROS even after normalising glucose levels, which can initiate an aggressive cycle of mtDNA damage leading to its mutation [73]. Methylglyoxal also exerts a rapid and irreversible effect on the TCA cycle enzymes, leading to the inhibition of whole respiration and decreased NADH production or leakage [74].

The sustained activation of AGE/RAGE-ROS triggers multiple cellular stress-sensitive pathways, such as PKC, NFκB, mitogen-activated protein kinase (MAPK) and signal transducer and activator of transcription 3 (STAT3), amplifying inflammatory and fibrotic responses in diabetic cardiomyopathy [75]. AGEs trigger ROS-dependent apoptosis in cardiomyocytes through PKCδ-mediated mitochondrial damage. A downstream effector of PKCδ, JNK, was found to be regulated by miR-210, which is known to play a cardioprotective role in mitochondrial ROS reduction [76,77]. AGE exposure in cardiomyocytes reduces miR-210 levels while upregulating JNK, contributing to IRS-1 inhibition and apoptosis [78].

#### 2.1.4. Protein Kinase C

PKC isozymes are a family of serine/threonine kinases, central enzymes regulating cell growth, hypertrophy and signal transduction in the heart, including individual ion channels, gap junctions and exchangers [79,80]. They reside with different distributions between atria (PKC-ζ and PKC-δ) and ventricles (PKC-α, PKC-βI and PKC-βII), with PKC-ε and PKC-λ evenly distributed [81]. The hyperactivation of PKC in diabetic cardiomyopathy is connected to mitochondrial dysfunction [82], hypertrophy [83], arrhythmias [80] and fibrosis [84].

The activation of PKC typically requires diacylglycerol (DAG) as a cofactor (Figure 3), which is mainly synthesised during de novo lipid biosynthesis [24]. Hyperglycaemia increases the availability of the glycolytic intermediate glyceraldehyde-3-phosphate (GAP), a DAG precursor, due to the inhibition of the glycolytic enzyme glyceraldehyde-3-phosphate dehydrogenase (GAPDH) by the PARylation process [85]. To detect the gradually increasing DNA damage due to ROS production, poly-ADP-ribose polymerase-1 (PARP-1) is recruited to detect and repair DNA strand breaks by the poly-ADP-ribosylation (PARylation) process to repair oxidative damage on the cellular DNA. However, as GAPDH can be a target for PARylation, its inhibition limits glycolytic metabolism, accumulating GAP and other glycolytic intermediates. Intracellular ROS from these pathways can activate PKC by oxidising its regulatory domain, even in the absence of DAG, entering a vicious cycle of positive feedback [23,86].

Certain isoforms of PKC affect cardiomyocytes differently and sometimes with opposing actions. PKC-θ has been proven to be cardioprotective in ischemic events. as cardiac injury markers and the apoptosis rate following hypoxia in PKC-θ gene deletion cardiomyocytes were much higher than in wild-type cardiomyocytes [87]. However, under chronic diabetic influence, persistent overexpression is associated with cardiac hypertrophy and fibrosis, believed to be due to PKC-MAPK pathway activation [88].

AGE treatment of cardiomyocytes showed dose- and time-dependent PKC-δ activation, leading to ROS generation, mitochondrial fragmentation and apoptosis, which can be prevented by inhibiting PKC-δ via PKC-δ-specific siRNA [29]. In human skeletal muscle, DAG-mediated PKC-θ activation inhibited the Akt pathway by phosphorylating IRS-1, blocking IRS1 tyrosine phosphorylation and downstream activation of the PI3K/Akt cascade [52,89].

#### 2.1.5. Hexosamine Pathway

Hyperglycaemia also increases glucose flux into the hexosamine pathway, which normally utilises 2–5% of intracellular glucose [90]. This pathway is induced during hypoxic and ischemic conditions, temporarily improving mitochondrial function and Ca^2+^ uptake and conferring cardiac protection against hypertrophy and apoptosis [91].

However, the chronic diabetic milieu shifting glucose into this pathway results in the conversion of glycolytic intermediate Fructose-6-phosphate into Glucosamine-6-phosphate, then into UDP-GlcNAc (uridine diphosphate-N-acetylglucosamine). This leads to excessive O-GlcNAcylation via enzymatic O-linkage attachment of specific serine/threonine residues of numerous cytosolic and nuclear proteins. *O*-GlcNAc transferase (OGT) is the enzyme that catalyses the reversible *O*-GlcNAcylation process, whereas *O*-GlcNAcase removes the O-GlcNAc residues [92].

This process is a posttranslational protein modification that affects multiple cellular pathways. Interference with the cardiac function was measured in altered Ca^2+^ uptake, mitochondrial fragmentation, systolic dysfunction and insulin insensitivity [28,93]. Key components of insulin signalling, such as the insulin receptor and Akt, can be O-GlcNAcylated at the same phosphorylation site, worsening insulin resistance and diminishing the cardioprotective effect [94,95]. Mitochondrial proteins can also be potential targets for O-GlcNAc modification. Mitochondrial acetaldehyde dehydrogenase 2 (ALDH2) is a crucial enzyme that protects the heart, mitigates post-ischemic oxidative damage and prevents cardiomyocyte apoptosis. Its activity is inversely related to ischemic infarct because it facilitates the detoxification of aldehydes produced from lipid peroxidation under high ROS conditions [96]. However, hyperglycaemic milieu increases ALDH2 *O*-GlcNAc modification, resulting in toxic aldehyde buildup and cell apoptosis via mPTP formation and cytochrome *c* release [97] Increased O-GlcNAcylation of mitochondrial proteins is linked to diminished complex I, III and IV activity and reduced mitochondrial Ca^2+^ and cellular ATP levels [98]. Reducing excessive O-GlcNAc modification will enhance mitochondrial function, normalise complex activity and restore mitochondrial Ca^2+^ and cellular ATP levels [99].

### 2.2. Mechanisms of Lipotoxicity and Ceramide-Mediated Dysfunction

#### 2.2.1. Metabolic Shift Towards Fatty Acid Oxidation

Circulating levels of FAs in the blood largely determine their cardiomyocyte uptake, which connects heart diseases to high-fat diets [100]. These FAs are mainly transported into the cell by CD36, where they are converted into fatty acyl-CoA for β-oxidation in the mitochondria or further converted to triacylglycerol for storage [101]. The PI3K-Akt pathway promotes the translocation of CD36 to the cell membrane to transfer lipids into the cytoplasm, and insulin’s ability to initiate this pathway is relatively reserved in diabetic cardiomyocytes; therefore, its effect is elevated with hyperinsulinemia [102]. CD36 overexpression is also promoted in diabetic mice through microRNA 320, which directly acts on its transcription [103].

Peroxisome proliferator-activated receptors (PPARs), the lipid metabolism transcription factors, are activated by the presence of FA in the cytoplasm [104]. Transgenic mice with a genetic overexpression of PPARγ revealed increased lipid oxidation and storage with distorted mitochondrial architecture in cardiomyocytes [105]. The activation of PPARα facilitates the inhibition of pyruvate dehydrogenase kinase, a key enzyme for glycolysis, therefore decreasing glucose oxidation [106]. In addition to the effects of increased FAO, when FA uptake overwhelms the oxidative capacity of the mitochondria, lipids accumulate intracellularly despite the limited storage capacity of the cardiac cells [40].

Medium and short-chain acyl-CoA can pass through the mitochondrial membrane. In the case of long-chain FAs such as palmitate, carnitine combines with the acyl group of long-chain acyl-CoA to form long-chain acylcarnitine through carnitine palmitoyltransferase-1, which is located in the outer mitochondrial membrane [107]. After being transported into the mitochondria via carnitine acylcarnitine translocase, long-chain acylcarnitine is broken down into long-chain acyl-CoA and carnitine by carnitine palmitoyltransferase-2 in the inner mitochondrial membrane [108]. The buildup of free long-chain fatty acids prevents the oxidation of medium and short-chain fatty acids, leading to higher concentrations of medium and short-chain acyl-CoA in the cytosol and mitochondria [109].

The accumulation of intermediate metabolites like acylcarnitines is associated with mitochondrial lipotoxicity in murine hearts [110] and was found to have elevated plasma levels in diabetic patients [109]. The increase in FA uptake, especially palmitate, and the subsequent overload of acyl-CoA in the cytosol results in excessive recycling into ceramides. These are signalling lipids acting as second messengers in stress responses to initiate mitophagy, which, at abnormally high levels, are associated with mitochondrial dysfunction, ROS formation and apoptosis [53,111,112].

#### 2.2.2. Ceramides-Cardiolipin Interactions

**Figure 4:**
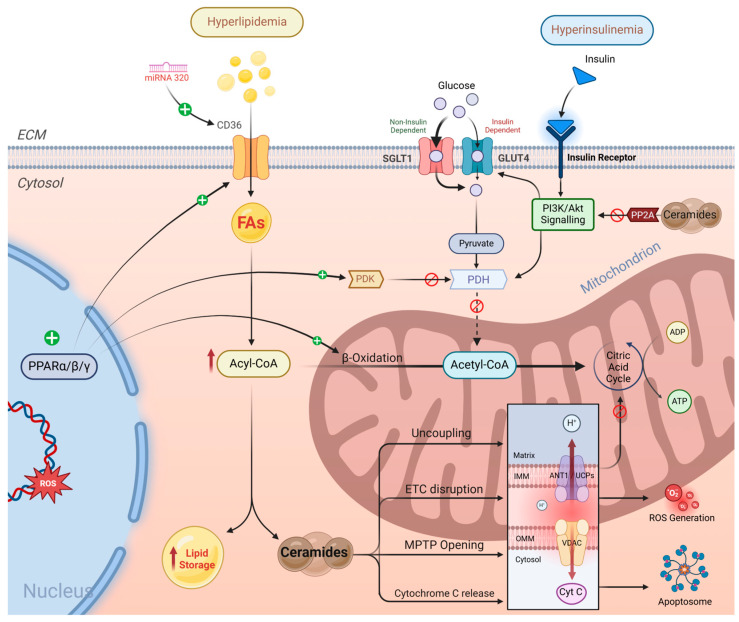
Mechanisms of lipotoxicity in diabetic cardiomyopathy. Normally, insulin triggers GLUT4 translocation and glucose uptake into the cell to be utilised by converting to pyruvate, then into acetyl-CoA that enters the citric acid cycle to generate ATP. Insulin resistance causes a metabolic shift to fatty acid metabolism. This shift leads to the overexpression of PPARs that upregulate lipid uptake and oxidation and downregulate glucose oxidation. The subsequent increase in the generation of ROS leads to disrupted pathways and destruction of DNA and the cellular membrane. Mitochondrial membrane disruption may lead to leakage of cytochrome C into the cytosol, initiating the apoptotic cascade. Altered Ca^2+^ handling occurs as sarcoplasmic receptors are desensitised, releasing a decreased flux of Ca^2+^ ions and weakening the cardiomyocyte contraction. Created with BioRender.com.

Cardiolipin, a phospholipid component of the inner mitochondrial membrane (IMM), is primarily affected as ceramides can integrate into mitochondrial membranes and disrupt cardiolipin organisation [113]. Cardiolipin forms the IMM cristae, stabilises the mitochondrial membrane potential (Δψm) and binds to IMM complexes, holding them in supercomplexes that crucially maintain the mitochondrial electron transport chain (ETC) and anchor cytochrome c to the IMM [114]. By disrupting cardiolipin, ceramides trigger several mechanisms, as shown in Figure 4: (a) damaging mitochondrial membranes and increasing membrane permeability, thereby inducing uncoupling and proton leakage; (b) depolarising the mitochondrial membrane potential, leading to mitophagy proteins accumulating on the outer membrane and increasing fragmentation [115]; (c) destabilising mitochondrial ETC and reducing electron flow, thereby impairing ATP production [116]; and (d) opening mPTP by binding and modulating its inner and outer membrane components, thereby activating apoptosis via cytochrome c release [117].

Cultured iPS-cardiomyocytes treated with high-fat media suffered from high ROS production, mitochondrial dysfunction, excessive mitophagy and eventual apoptosis due to ceramide accumulation [53]. They revealed high levels of CERS2 (ceramide synthase 2), a key enzyme involved in synthesising very long-chain ceramides, confirming a previous conclusion in clinical and animal studies that a lipotoxic environment and ceramide deposition are linked to cardiac dysfunction [118,119,120]. Ceramides also exacerbate insulin resistance by activating PP2A, which inhibits the PI3K/Akt pathway [121].

## 3. Cellular and Molecular Drivers of Cardiac Dysfunction

### 3.1. Calcium Homeostasis

#### 3.1.1. Regulation of Intracellular Ca^2+^

**Figure 5:**
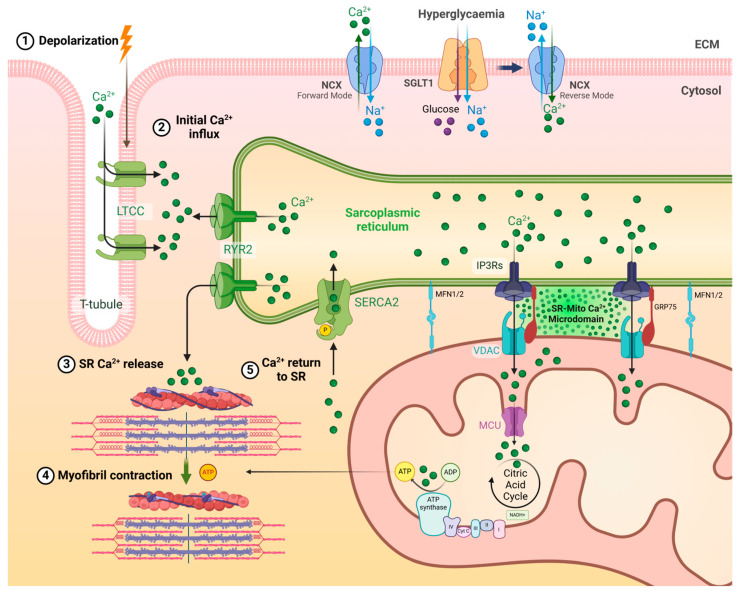
Cellular and mitochondrial calcium handling in cardiomyocytes. (1) Cardiac muscle contraction begins as action potential travels through sarcolemma, triggering (2) the initial Ca^2+^ influx via LTCCs in the T-tubule. (3) The initial rise in cytosolic Ca^2+^ stimulates SR Ca^2+^ release through RYR2. (4) Ca^2+^ binds to myofilaments, enabling ATP-dependent contraction. (5) For muscle relaxation, Ca^2+^ is returned to the SR via SERCA2 to restore homeostasis. Additionally, Ca^2+^ is transferred to mitochondria via VDAC, taking advantage of the SR-mitochondria Ca^2+^ microdomain. MCUs are highly selective for Ca^2+^ entry into the mitochondrial matrix, where they enhance ATP production via the TCA cycle and electron transport chain. Dysregulated Ca^2+^ handling in hyperglycaemia contributes to contractile dysfunction and metabolic alterations. One example is the increased Na^+^ influx through SGLT1, activating the reverse mode of the NCX leading to excess intracellular Ca^2+^ levels. Abbreviations: LTCC, L-type calcium channel; RYR2, ryanodine receptor 2; SERCA2, sarcoplasmic/endoplasmic reticulum Ca^2+^-ATPase; VDAC, voltage-dependent anion channel; MCU, mitochondrial calcium uniporter; SGLT1, sodium-glucose cotransporter 1; NCX, sodium-calcium exchanger. Created with BioRender.com.

The preservation of Ca^2+^ homeostasis is fundamental for maintaining optimal heart function, including regulating myocardial metabolism, contractility and signal transduction pathways [122]. A minor influx of Ca^2+^ drives cardiac contraction due to the action potential travelling through the sarcolemma and activating voltage-gated L-type Ca^2+^ channels (LTCC) [123]. This influx triggers Ca^2+^-induced Ca^2+^ release (CICR), activating the type-two ryanodine receptor (RyR2) located in the sarcoplasmic reticulum (SR) membrane, thereby releasing a larger amount of Ca^2+^ from the SR into the cytosol [124]. The cytosolic Ca^2+^ binds to cardiac Troponin C, inducing actin-myosin filament sliding and ultimately causing muscle contraction, as shown in (Figure 5). Rapid mitochondrial Ca^2+^ uptake and binding in the matrix is essential to generate ATP for energy contraction, as well as buffering cytosolic Ca^2+^ to reduce high concentration peaks [125]. Studies measuring transience during systole found that the amount of cytosolic Ca^2+^ rise was approximately ~75 μM per litre of cytosol, with ~1% taken up by mitochondrial transporters [126,127]. The matrix Ca^2+^ concentration under β-adrenergic stimulation could reach up to 800 nmol/L over repeated cycles [128].

For cardiac muscle relaxation, Ca^2+^ in the cytosol has to be pumped back into the SR through Sarco/Endoplasmic Reticulum Ca^2+^-ATPase (*SERCA*) channels located on the SR membrane, as well as some outflux through the cell membrane via the sodium-Ca^2+^ exchanger (NCX) and the plasma membrane Ca^2+^-ATPase transporter [129]. Normally, NCX operates in forward mode, extruding one Ca^2+^ ion out of the cell in exchange for three sodium ions, thereby helping to remove excess Ca^2+^ from the cytosol during relaxation [130]. Under hyperglycaemic upregulation of SGLT1, which co-transports glucose and sodium into the cell, high sodium levels are incited, triggering reverse-mode NCX activity and increasing Ca^2+^ influx that disrupts excitation–contraction coupling and impairs relaxation [131].

Several cellular compensatory mechanisms can shift Ca^2+^ in and out of the cell to maintain Ca^2+^ homeostasis, which presents heterogeneous findings regarding cytosolic Ca^2+^ levels in diabetic cardiomyopathy [132]. However, evidence indicates that Ca^2+^-related contractile dysfunction results from alterations in cytosolic Ca^2+^ cycling, causing a decrease in Ca^2+^ transient amplitude, lower systolic Ca^2+^ levels or higher diastolic Ca^2+^ levels [133,134,135].

Diabetic conditions affect Ca^2+^ transience in multiple ways. Oxidative stress reduces both ryanodine receptor sensitivity and receptor number [136]. Diabetic mice hearts revealed reduced SERCA2a expression, a slowdown in SR reuptake and increased Ca^2+^ SR leakage [123,134]. These dysregulations manifest as increased action potential duration and prolonged diastolic relaxation time, which results in systolic and diastolic dysfunction [137]. Methylglyoxal, the hyperglycaemic byproduct, chemically reacts with and forms adducts on both RYR2 and SERCA2a, disrupting cytosolic Ca^2+^ release and uptake [138]. Another hyperglycaemic effect on Ca^2+^ receptors is the increased O-GlcNAcylation of phospholamban (PLN), the Ca^2+^ pump SERCA2 regulator, leading to altered transient ion Ca^2+^ and delayed Ca^2+^ peaks [99]. The excess PKCε was linked to arrhythmias because of hyperphosphorylation of RYR2, an effect that triggers store-overload-induced Ca^2+^ release (SOICR) [139]. This phenomenon is associated with Ca^2+^ dysregulation and arrhythmias, where the RYR2 threshold to SR Ca^2+^ decreases and initiates a spontaneous Ca^2+^ release through RYR2 [140].

#### 3.1.2. Mitochondrial Ca^2+^ Cycling

Mitochondria in cardiomyocytes appear in electron microscopy to be located in close proximity to the SR, tethered by MFN1/2, where the Ca^2+^ channels RYR2 and the voltage-dependent anion channel (VDAC) are clustered (Figure 5). This channel cluster creates Ca^2+^ microdomains, where the Ca^2+^ concentration exceeds average total cell levels, rising as high as 20 μM, allowing higher Ca^2+^ uptake in mitochondria privileged with these microdomains [141,142]. Ca^2+^ enters mitochondria via VDAC in the outer mitochondrial membrane (OMM), which is highly permeable to Ca^2+^ ions [143]. On the other hand, the IMM tightly controls Ca^2+^ entry. This regulation is managed by the mitochondrial Ca^2+^ uniporter (MCU), which transports Ca^2+^ ions across the IMM based on the mitochondrial membrane potential (Δψm). Altered expression of MCU in the hyperglycaemic conditions in murine diabetic hearts and cultured neonatal cardiomyocytes negatively affected mitochondrial Ca^2+^ uptake, glucose oxidation, ATP production and cardiac contractility [144,145].

Ca^2+^ is crucial in regulating key dehydrogenase enzymes in the TCA cycle, enhancing metabolic flux and ATP production. Amongst others is PDH, of which activity is influenced by acetyl-CoA/CoA and NADH/NAD^+^ ratios, but its activator, Pyruvate dehydrogenase phosphatase (PDP), has a catalytic subunit (PDP1c) that Ca^2+^ specifically activates [146]. Beyond the TCA cycle, research has identified a regulatory role for Ca^2+^ in oxidative phosphorylation. In the presence of Ca^2+^, IF1, the inhibitory protein of Complex V, is phosphorylated, reducing its inhibitory effect on ATP synthase and allowing increased ATP production [147]. Ca^2+^ treatment in isolated skeletal muscles was able to activate the entire muscle oxidative phosphorylation cascade; specifically, the conductance of Complex IV was increased by 2.3-fold, Complexes I and III by 2.2-fold and ATP production/transport by 2.4-fold [148]. Diabetic alteration in Ca^2+^ transience may lead to excess cytosolic Ca^2+^ being taken up by mitochondria via the MCU, leading to mitochondrial Ca^2+^ overload and impaired ATP production [149]. Excess Ca^2+^ in the mitochondrial matrix may increase the production of NADH through PDH, which overloads the mitochondrial ETC, leading to increased ROS production, as will be discussed next.

### 3.2. Oxidative Stress

#### 3.2.1. ROS Generation in Diabetic Cardiomyocytes

Physiologically, ROS production in cardiomyocytes mediates several biological pathways, all while ROS scavengers balance their presence. Cardiomyogenesis is induced by cyclic strain that relies on ROS-dependent integrins and the subsequent activation of the PI3K/Akt signalling pathway [150]. Localised ROS production contributes to the stretch-induced enhancement of cardiac contractile activity. It also sensitises RYR2s in the SR, optimising their Ca^2+^ sensitivity [151].

Under normal conditions, these free radicals can be neutralised by antioxidant systems. However, in diabetic conditions, a severe imbalance between ROS generation and antioxidant capacity manifests as oxidative stress, which largely contributes to the deterioration of mitochondrial function and cell death [152]. Several factors, such as hyperglycaemia, hyperlipidaemia, insulin resistance and enhanced mitochondrial uncoupling, lead to excessive ROS production in diabetic cardiomyopathy [153]. The family of ROS is associated with oxidative damage to proteins, nucleic acids, lipids and cellular structures via a variety of mechanisms, including oxidation and the alteration of signalling pathways [154]. Oxidative stress is exacerbated by conditions associated with diabetes, such as metabolic syndrome, hypertension, hyperglycaemia, obesity and dyslipidaemia [155].

Furthermore, oxidative stress can lead to mutations that compromise mitochondrial respiratory chain function, further exacerbating ROS production [15]. In endothelial cells, ROS-mediated damage impairs NO bioavailability, leading to endothelial dysfunction, reduced vasodilation and compromised coronary blood flow [156]. Due to the high metabolic activity and low antioxidant availability in the heart, it is more susceptible to oxidative damage [157]. Superoxide, being the dominant ROS in the heart, is mainly produced by the mitochondrial electron transport chain and NADPH oxidases (NOXs) [158]. Other sources include xanthine oxidase and uncoupled nitric oxide synthases [159].

#### 3.2.2. Mitochondrial ROS Production

**Figure 6:**
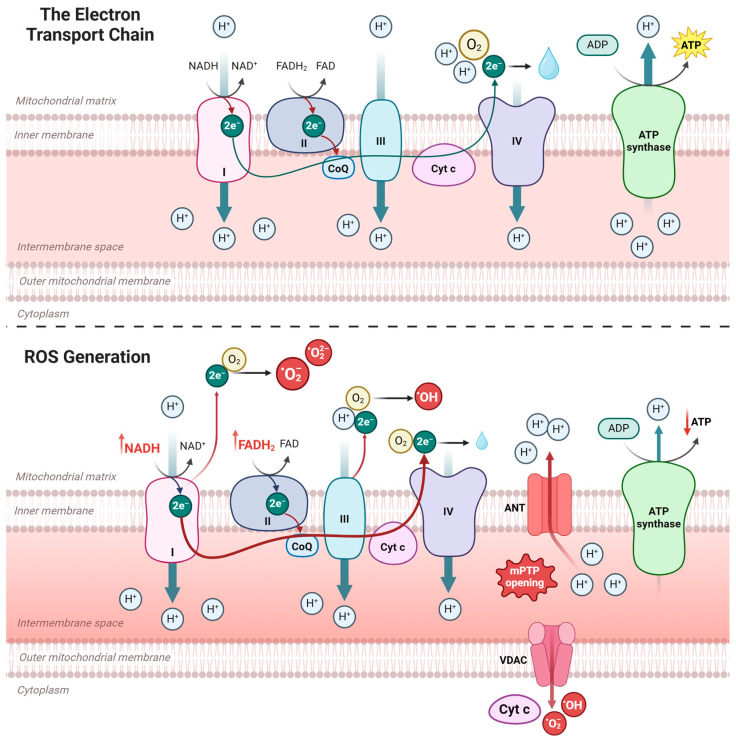
Mitochondrial ROS generation. Electrons derived from NADH and FADH_2_ are transferred through Complexes I to IV. Under normal conditions, Complex IV efficiently transfers electrons to oxygen to produce water. However, under diabetic conditions, excess electron flow can lead to electron leakage, particularly at Complexes I and III, as premature reactions with O_2_ generate superoxide, hydroxyl radicals and hydrogen peroxide. ROS disrupt mitochondrial integrity, oxidise membrane lipids and promote mPTP opening. Once the mPTP opens, mitochondrial homeostasis is lost, allowing the uncontrolled movement of protons (H^+^) into the matrix, causing dissipation of membrane potential (Δψm) and failure of ATP production. Additionally, key apoptotic factors such as cytochrome c leak into the cytosol. Created with BioRender.

It is important to consider the role of the mitochondrial ETC in oxidative stress (Figure 6). Physiologically, the ETC functions by accepting electrons donated by co-enzymes NADH and FADH_2_ that then proceed to Complex I of the ETC (NADH ubiquinone reductase), Complex II (succinate dehydrogenase), Complex III (Ubiquinol-cytochrome c reductase) and Complex IV (cytochrome c oxidase) before finishing as oxygen via Complex V (F_0_F_1_ ATP synthase), all of which occur alongside the electron transfer across the inner membrane and, thus, ATP generation.

Mitochondrial ROS production occurs naturally when excess flow of NADH and FADH2 to the respiratory chain prevents electron transfer at complex III due to IMM hyperpolarisation. Suppose Complex IV cannot efficiently transfer electrons to oxygen. In that case, electrons build up, leading to premature reactions with molecular oxygen and the formation of superoxide and other ROS-like superoxides, hydrogen peroxide and hydroxyl radicals [160]. In addition to the mitochondria, the SR protein folding is an oxidative process that also generates considerable amounts of ROS [161]. Superoxide interaction with nitric oxide forms a more powerful oxidant, peroxynitrite, capable of inducing the formation of mPTPs and initiating apoptosis [162,163].

Mitochondrial DNA (mtDNA) is further susceptible to oxidative damage induced via excessive ROS associated with T2DM; enhancements in ROS were further associated with an accumulation of mtDNA mutations, thus resulting in an increase in mitochondrial damage, reducing the antioxidant presence and affecting ATP generation and Ca^2+^ homeostasis [155].

Another factor leading to impaired electron transfer is the upregulation of the mitochondria uncoupling protein (UCP) activity, specifically UCP2 and UCP3. Under oxidative stress conditions, ROS damages mitochondrial ETC, opening mPTP on the IMM. This allows electrons to pass into the mitochondrial matrix without generating ATP [163]. This “uncoupling” of the ETC can further generate more superoxide, disrupting the mitochondria’s proton gradient. Therefore, oxygen consumption increases and ATP production decreases, a situation already worsened by excessive FOA as UCP3 expression is enhanced by PPARα [164,165].

#### 3.2.3. Antioxidant Pathways in ROS Defence

Several antioxidant pathways, such as AMPK and the PI3K/Akt Pathway, play their roles in antioxidant defence by activating downstream target antioxidant enzymes. These include superoxide dismutases (SOD1/2/3), which convert superoxide radicals into hydrogen peroxide. Catalase (CAT) and glutathione peroxidases (GPx1, GPx4) further neutralise hydrogen peroxide by converting it into water and oxygen [34,166].

**Figure 7:**
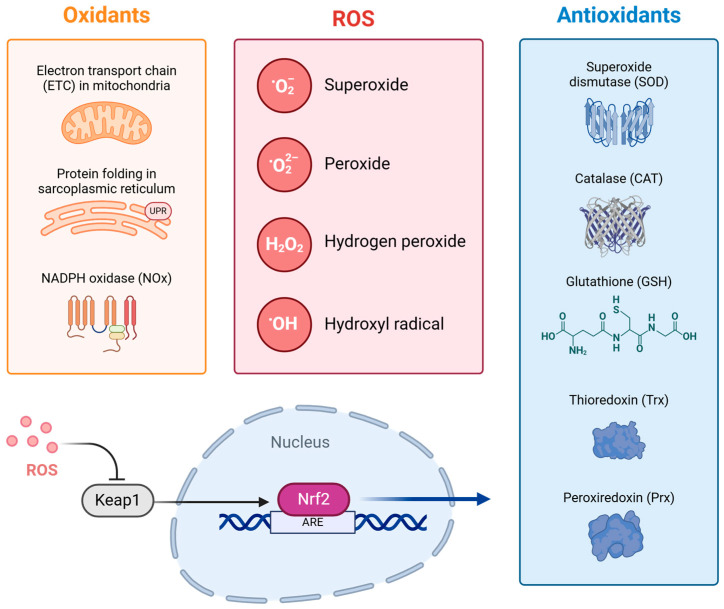
The redox balance in cardiomyocytes, highlighting the interplay between oxidants, reactive oxygen species (ROS) and antioxidants. ROS generation comes from the electron transport chain in mitochondria, protein folding in the sarcoplasmic reticulum and UPR activation and NADPH oxidases. The ROS family includes superoxide, peroxide, hydrogen peroxide and hydroxyl radicals, which disrupt KEAP1-NRF2 binding and release NRF2 that promotes transcription of antioxidant genes. The antioxidant defence mechanisms involve superoxide dismutase, catalase, glutathione, thioredoxin and peroxiredoxin, which neutralise ROS and maintain cellular homeostasis. This regulation is critical for protecting cardiomyocytes, particularly in diabetic iPS-CMs, where oxidative stress contributes to mitochondrial dysfunction and impaired contractility. Created with BioRender.com.

A key master regulator of cellular antioxidation is Nuclear Factor Erythroid 2-Related Factor 2 (NRF2), not to be confused with the nuclear respiratory factor 2 with a similar acronym, which regulates the cellular responses against environmental stresses by promoting the expression of detoxification and antioxidation enzymes [167,168]. Under normal conditions, NRF2 is bound to Kelch-like ECH-associated protein 1 (KEAP1), which facilitates the degradation of NRF2 through ubiquitination. During episodes of oxidative stress, ROS modifies KEAP1 and dissociates it from NRF2. This allows NRF2 to translocate to the nucleus and bind to Antioxidant Response Elements (AREs) in the DNA to activate its target genes, as shown in Figure 7 [169].

NRF2 regulates the expression of multiple antioxidant genes, including the aforementioned SOD, CAT and GPx [170]. In addition, NRF2 activates thiol-based redox compounds such as thioredoxin (Trx), glutaredoxin (Grx) and peroxiredoxin (Prx). Trx functions as an antioxidant by reducing oxidised proteins, and Prx detoxifies hydrogen peroxide, hydroperoxides and peroxynitrite [171]. Grx1 reactivates glutathione and supports ROS detoxification by interacting with Trxs and Prxs. It also regulates factors such as Ras and NFκB, which are related to apoptosis and inflammation, respectively [172].

Another redox function of NRF2 is redirecting glucose into intermediary metabolic pathways, specifically the pentose phosphate pathway, by upregulating its rate-limiting enzymes. This pathway reduces cardiac dysfunction under stress by generating NADPH and nucleotides, which prevent DNA damage and the cardiomyocytes’ death [173].

However, chronic stimulation of NRF2 in response to oxidative stress was found to deteriorate insulin sensitivity [174]. Due to its complex signalling network, the duality in the effect of NRF2 activation depends on many factors, including the disease processes, the timing of its response and the location in bodily tissue [169]. Levels of NRF2 were found to be either low or high in diseases such as diabetes, prostate cancer, inflammatory diseases and cancer, depending on the disease stage. Therefore, with activators and inhibitors, NRF2 has been used as a therapeutic target for many diseases.

#### 3.2.4. NADPH Depletion

NADPH is essential for glutathione reductase to maintain this antioxidation process, which reduces oxidised glutathione back to reduced glutathione [175]. In hyperglycaemic conditions, NADPH may be depleted by being consumed in the polyol pathway or by NADPH oxidase. In the polyol pathway, aldose reductase reduces excess glucose to sorbitol using NADPH as a cofactor [27]. Also, the conversion of sorbitol into fructose consumes NAD^+^ and eventually disrupts the NADH/NAD^+^ balance [176]. High levels of NADH overload mitochondrial complex I, causing electron leakage and ROS production, and suppress glycolysis by inhibiting the pyruvate dehydrogenase complex [177]. The overexpression of aldose reductase resulted in increased atherosclerosis in diabetic mice and inhibited glutathione regeneration [178]. On the other hand, the inhibition of aldose reductase, as a rate-limiting enzyme, was shown to prevent diabetic complications [179].

In addition to the polyol pathway, a major key contributor to excessive ROS production is the overexpression of specific NADPH oxidase isoforms (NOX2 and NOX4) [152]. Under normal conditions, low levels of NOX-derived ROS serve as signalling intermediates essential for different cell activities, such as cell adaptation, migration and proliferation of endothelial cells [180]. However, as diabetic cardiomyopathy progresses, NOX-derived ROS production abnormally exceeds the antioxidative defence capacity.

Elevation in NOX2 protein expression was extensively noted in the literature, whether in acute [181] or chronic hyperglycaemia [182]. PCK activation via the accumulation of glycolytic intermediates, as a result of SGLT1′s insulin-independent glucose influx, leads to PKC-induced NOX2 upregulation through the translocation of Rac1, an NADPH-oxidase subunit, to the cell membrane [183]. On the other hand, reducing NOX activity using Canagliflozin [184] or Phlorizin [181] (SGLT1 inhibitors) resulted in anti-inflammatory and anti-apoptotic effects in the human myocardium via AMPK-mediated suppression of membrane translocation of Rac1, a key step in NOX2 activation [184]. They also help restore the balance between glycolysis and mitochondrial glucose oxidative phosphorylation.

#### 3.2.5. PARP-1 and NAD^+^ Depletion

The oxidative DNA damage sensor enzyme PARP-1 detects and repairs DNA strand breaks using the PARylation process [58]. PARP-1 is the most abundant isoform of the PARP enzyme family, regulating multiple physiological cellular functions, including DNA repair, gene transcription and genomic stability [185]. To repair DNA, PARP-1 catalyses the NAD^+^ to form long branches of ADP-ribose polymers on the target proteins, acting as a scaffold to recruit other DNA repair proteins [58,186]. Excessive activation of this enzyme in hyperglycaemic conditions depletes intracellular NAD^+^ and ATP, causing energy deficiency and cytotoxicity [187]. The inhibition or deletion of PARP-1 in mice cardiomyocytes decreased ROS-induced apoptosis by downregulating cleaved caspase-3 and caspase-9 alongside activating the insulin-like growth factor 1 receptor (IGF-1R)/Akt pathway, which plays a significant role in pro-survival and anti-apoptotic signalling [188].

Along with the previous sources of oxidative stress, endoplasmic reticulum stress releases a considerable portion of ROS production, which not only impairs energy production but also triggers a cascade of cellular damage through oxidative stress and altered metabolic pathways.

### 3.3. Sarco/Endoplasmic Reticulum Stress

As discussed earlier, SR as a specialised domain of ER, plays a critical role in cardiomyocyte Ca^2+^ transient balance since protein folding relies on a tightly controlled Ca^2+^ environment for the regulation of folding chaperones and the UPR [189].

The ER folds proteins into their functional three-dimensional structures, assisted by forming disulfide bonds through the collaboration of resident protein disulfide isomerases (PDI), endoplasmic reticulum oxidoreductin-1 (ERO1) and glutathione [190]. The oxidative folding process generates considerable amounts of ROS and is counterbalanced by the glutathione pool [161].

Oxidative stress from diabetic mitochondrial dysfunction modifies proteins, making them more prone to misfolding [191]. With increased protein production due to hyperglycaemia and mTOR activation, improper disulfide bond formation causes the accumulation of misfolded proteins in the ER lumen, resulting in ER stress [192]. As a compensatory measure, the oxidative folding becomes hyperactivated to fix these incorrect bonds, generating more ROS, which, in turn, disrupts the PDI function, further accumulating misfolded proteins [193].

ER stress activates UPR to restore normal function by halting protein translation and enhancing the folding process pathways [194]. Typically, the unfolded transmembrane ER proteins are bound to intraluminal ER chaperone glucose-regulated protein 78 (GRP78) to prevent their aggregation. However, during ER stress, a large accumulation of unfolded proteins in the ER lumen requires GRP78 to dissociate, triggering the UPR. Despite UPR being a protective mechanism, chronic activation contributes to cardiomyocyte apoptosis, inflammation and cardiac fibrosis [195].

The unfolded protein response (UPR), which is elicited by ER stress, activates three transmembrane sensors by dissociating from GRP78: PERK (Protein kinase RNA-like ER kinase), IRE1 (Inositol-Requiring Enzyme 1) and ATF6 (Activating Transcription Factor 6) [196]. PARP16 also correlates with the functional activation of ER stress sensors PERK and IRE1α during the UPR [197].

PERK activation leads to a temporary reduction in protein synthesis to alleviate ER stress. Under diabetic conditions, prolonged activation induces ROS production and activates C/EBP homologous protein (CHOP), a key mediator of ER stress-induced apoptosis [54]. CHOP downregulates anti-apoptotic Bcl-2 members and increases the expression of pro-apoptotic proteins BAX and BAK. Activation of the PERK-CHOP pathway also worsens insulin resistance by inhibiting PI3K and Akt [198].

IRE1 is a dual-function kinase and endoribonuclease that mediates adaptive and maladaptive responses under ER stress. The chronic activation of mTORC1 serves ER stress-triggered apoptosis via selective activation of the IRE1–JNK pathway, as chronic IRE1 activation leads to JNK recruitment associated with NFκB-induced inflammation, apoptosis and myocardial fibrosis [55]. The overexpression of PARP16 exacerbated the hypertrophic responses and increased cardiomyocyte surface area and upregulation of the hypertrophic markers’ levels by activating the IRE1α-GATA4 pathway [197].

ATF6, a transcription factor activated under ER stress, upregulates GRP78 to improve protein-folding capacity and enhance cardiomyocyte growth and hypertrophy by promoting GATA4 activation [199]. While this pathway mainly promotes cell survival, prolonged ATF6 activation could contribute to elevated apoptotic signalling through its interaction with CHOP, which is associated with increased lipid metabolism and cardiac hypertrophy [200].

Chronic ER stress in diabetic mice activated multiple apoptotic pathways, including caspase-12, BAX and CHOP, resulting in the loss of cardiomyocytes and ventricular dysfunction [201]. Drug-mediated PI3K/Akt/mTOR activation was found to be protective and prevents myocardial apoptosis and excessive UPR [202]. However, as discussed earlier, the prolonged over-compensatory mTOR activation in diabetes may also deteriorate cardiac function.

ER stress induces diabetic cardiomyopathy pathogenesis by promoting apoptosis, inflammation and fibrosis through the PERK, IRE1 and ATF6 pathways. Acute UPR activation is adaptive, but chronic ER stress exacerbates cellular damage and leads to metabolic dysregulation and mitochondrial dysfunction.

## 4. Mitochondrial Dysfunction in Diabetic Heart

### 4.1. Mitochondrial Biogenesis

Mitochondria make up roughly 40% of the volume of cardiac cells, which is necessary to keep up with the high demand for cardiac function [203]. The mitochondrial quality control system is a complex network involving mitochondrial biogenesis to replenish the mitochondrial pool, fusion to merge mitochondrial content and fission to separate damaged mitochondria [204].

Many of these functions are regulated by PGC-1α, a master regulator coordinating mitochondrial biogenesis, mitochondrial dynamics and mitophagy. It modulates functional proteins in both transcriptional regulation and posttranslational modifications [205,206]. During ATP demand, PGC-1α is overexpressed, inducing the transcriptional expression of transcription factor NRF1 (nuclear respiratory factor 1) involved in the maintenance and biogenesis of mitochondria, which, in turn, stimulates the synthesis of TFAM (mitochondrial transcription factor A) that directly mediates mitochondrial DNA replication and transcription [206]. NRF1 regulates the gene expression of mitochondrial biogenesis, oxidative phosphorylation and proteins needed for mitochondrial DNA transcription and replication.

PGC-1α and NFκB mutually influence each other during inflammation. Normally, PGC-1α regulates pro-inflammatory cytokine levels by blocking NFκB transcriptional activity toward these target genes [207]. Diabetes is known to be a low-grade inflammatory state, which increases the expression of NFκB and downregulates PGC-1α expression [208]. The reduction in PGC-1α activity causes a decline in the translation of its antioxidant target genes, increasing oxidative stress and worsening the inflammatory response [208].

In the context of diabetes, reduced insulin sensitivity and chronic hyperglycaemia downregulate PGC-1α expression, leading to impaired mitochondrial biogenesis [209]. Long-chain ceramides, as a by-product of hyperglycaemia and dyslipidaemia, improve mitochondrial dysfunction by activating PP2A, which dephosphorylates PGC-1α [53]. Mice with PGC-1α deletion suffered cardiomyopathy at 17 weeks and died prematurely at 25 weeks [210]. Conversely, drug-mediated activation of the PPAR-γ/PGC-1α signalling pathway in diabetic rabbits enhanced the expression of NRF1 and TFAM, decreased oxidative stress and prevented cardiac remodelling [211]. Reduced mitochondrial biogenesis in the high energy-demanding cardiomyocytes results in a depletion of mitochondrial numbers and a decreased capacity for ATP production, which is essential for maintaining the contractile function of the heart. As the heart becomes less capable of meeting its energy demands, the contractile function deteriorates, leading to systolic and diastolic dysfunction.

### 4.2. Mitochondrial Dynamics

**Figure 8:**
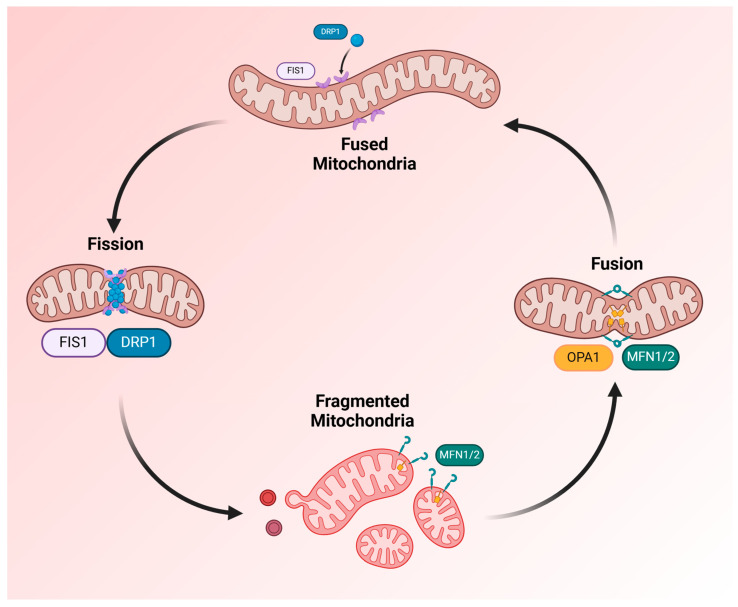
Mitochondrial dynamics. Balance between fusion and fission is essential to maintain mitochondrial function and adapt to cellular stress. Fusion allows mitochondria to mix their contents of mtDNA, proteins and metabolites, which helps them to mitigate damage and sustain energy production. Mitofusins (MFN1/2) on the outer mitochondrial membrane and OPA1 on the inner mitochondrial membrane mediate the fusion process. Fission, on the other hand, is primarily driven by Dynamin-related protein 1 (DRP1), which is recruited to the OMM by Fission protein 1 (FIS1) to initiate mitochondrial division. However, under pathological conditions such as diabetic cardiomyopathy, excessive fission over fusion leads to mitochondrial fragmentation, disrupting ATP production and increasing ROS generation. The hyperactivation of DRP1 and reduced MFN1/2 and OPA1 expression promotes mitochondrial dysfunction, cytochrome c release and apoptotic signalling, contributing to cardiac injury. Created with BioRender.com.

Mitochondrial fusion promotes the elongation of mitochondrial structures and increases the connectivity of the mitochondrial network, which increases their efficiency and oxidative capacity. Fusion allows mitochondria to mix their contents, including mtDNA, proteins and metabolites, helping to mitigate damage and sustain energy production [212]. Proteins regulating mitochondrial fusion are a group of dynamin-related large GTPases, including Mitofusin 1 and 2 (MFN1/MFN2) on the OMM and Optic atrophy 1 (OPA1), which regulates inner mitochondrial membrane fusion and cristae remodelling on the IMM (Figure 8). They mediate mitochondrial tethering and outer mitochondrial membrane fusion, allowing the formation of elongated tubular mitochondria [213,214].

The double knockout of MFN1/MFN2 genes in mice hearts resulted in a plethora of small, round mitochondria due to unopposed fission, inducing mitochondrial fragmentation dysmorphology, impaired respiration and rapidly progressive and lethal dilated cardiomyopathy [213]. However, isolated expression of one of them, MFN1 or MFN2, in mice hearts was sufficient for viability despite the greater prevalence of smaller spherical mitochondria [215]. The multiple effects of hyperglycaemia discussed earlier suppress the expression of OPA1 and MFN1 in cardiomyocytes while inducing mitophagy [216]. While observing rats developing diabetes over 4, 8 and 12 weeks, their cardiomyocytes suffered a gradual decline in MFN2 with a reduction in SOD activity, developing decreased antioxidant ability, mitochondrial fragmentation and progressive apoptosis [217].

Mitochondrial fragmentation occurs when the normally elongated and interconnected mitochondrial network breaks into smaller, discrete fragments. Fission enables mitochondrial quality control by promoting the segregation of damaged mitochondria for degradation via mitophagy. While mitochondrial fission is necessary for removing damaged mitochondria, excessive fragmentation is detrimental to cellular function [218].

Fission is regulated by cytoplasmic Dynamin-related protein 1 (DRP1) and its receptors, mitochondrial fission protein 1 (FIS1), mitochondrial fission factor (MFF) and mitochondrial dynamics proteins 49 (MiD49) and (MiD51). These proteins normally fragment the mitochondria to permit an even distribution and promote the segregation of damaged parts [31,219]. Damaged mitochondria are eliminated through a specific autophagic process called mitophagy, controlled by phosphatase and tensin homolog-induced putative kinase 1 (PINK1) and Parkin RBR E3 ubiquitin-protein ligase (Parkin) pathways [220]. In cardiac cells with knocked-down DRP1, a reduction in PINK1 and Parkin leads to increased cell death due to undegraded dysfunctional mitochondria initiating apoptosis [221]. Mitophagy is critical to maintaining mitochondrial quality control, but in diabetic cardiomyopathy, its excessive rate of mitochondrial clearance leads to a severe reduction in their number [222].

In cultured cardiomyocytes, it was shown that insulin plays a role in regulating mitochondrial dynamics by stimulating mitochondrial fusion and increasing OPA1 protein levels through the Akt-mTOR-NFκB signalling pathway [223]. Drug-mediated inhibition of NFκB leads to a redundant effect of insulin on OPA1 as NFκB acts downstream of the Akt-mTOR pathway.

Cardiomyocytes in mice with mutated OPA1 suffered late-onset cardiomyopathy with antioxidant transcription deficiency, increased ROS production and mitochondrial dysfunction [224,225]. In contrast, when diabetic mice were compared to control mice, the key fission regulator protein DRP1 was significantly increased in their cardiomyocytes, along with a significant decrease in OPA1 [226]. The genetic overexpression of DRP1 in mice cardiomyocytes also exhibited increased ROS production, mitochondria fragmentation and insulin resistance development [227].

Diabetic conditions have multiple pathways that induce mitochondrial fragmentation through DRP1 activation. DRP1 is shown to be modified by O-GlcNAcylation in murine cardiomyocytes, enhancing DRP1 recruitment to mitochondria [228]. When this increase is mediated by sustained hyperglycaemia, O-GlcNAcylation has been linked to the development of diabetic cardiovascular complications by increasing mitochondrial fission [229].

Human iPS-induced cardiomyocytes with an increased overproduction of ceramide also exhibited significant increases in DRP1 compared to the control [121]. Cyclin-dependent kinase 1 (Cdk1) and PKC-δ, which are increased in hyperglycaemia [230], played a critical role in ROS-driven phosphorylation of DRP1 Ser616 residue, promoting mitochondrial fission and cardiomyocyte death [82]. Orai1 (Calcium release-activated Ca^2+^ channel protein 1) activity was increased in the hearts of hyperglycaemic mice, activating DRP1 via the activation of ERK1/2, which phosphorylated DRP1 [216].

The subsequent fragmentation further leads to cellular stress and fibrosis in the heart tissue, contributing to diabetic cardiomyopathy and heart failure [231]. The fragmented mitochondria in endothelial cells may lead to compromised energy production and contribute to endothelial dysfunction. To counter an MI or ischemic event, anti-apoptotic pro-survival kinase signalling cascades such as PI3K, Akt and Erk1/2 occur. Moreover, Erk1/2 regulates cell proliferation by mediating cellular apoptosis via the inhibition of cytochrome-c-induced caspase activation. During mitochondrial fragmentation, the Bax protein speeds up apoptosis by triggering the release of mitochondrial cytochrome-c, which leads to mPTP formation and starts the process of cellular apoptosis.

The Bcl-2 and apoptosis protease activating factor (Apaf) family of proteins comprise the core apoptotic mechanism [232]. During mitochondrial cell death, several members of the pro-apoptotic Bcl-2 family, such as BAD, Bim, Bak and Bax, antagonise anti-apoptotic Bcl-2 family proteins to induce mitochondrial swelling and damage [233,234]. The release of cytochrome-c from the damaged mitochondria recruits caspase-9 and Apaf-1 to form an apoptosome complex [235]. Activated caspase-9 cleaves and activates caspase-3, which in turn cleaves the inhibitor of caspase-activated DNase (ICAD) to initiate ICAD induction of DNA laddering [236]. In addition, the release of the apoptosis-inducing factor (AIF) facilitates mitochondrial fragmentation and DNA damage [236,237].

The intricate balance of mitochondrial dynamics is critical for maintaining mitochondrial function. Its disruption can lead to mitochondrial fragmentation and also disrupt mitophagy, accumulating damaged mitochondria as a result.

### 4.3. Impaired Mitophagy

Parkin plays an essential role in protecting cardiac cells by mediating the selective removal of dysfunctional mitochondria through mitophagy. Parkin orchestrates the efficient degradation of damaged mitochondria, thereby preventing mitochondrial-driven cell death and maintaining cardiac function [220]. When ROS or mitochondrial damage occurs, mitochondria become depolarised and attract autophagy receptors. Once activated through PINK1, Parkin is recruited to the damaged mitochondria, where it ubiquitinates various outer mitochondrial membrane proteins, including VDAC, MFN1/2 and MFF [238].

Mitophagy targets damaged or dysfunctional mitochondria after they are labelled with receptors and ligands, SQSTM1/p62 (sequestosome 1) and ubiquitin by Parkin and PINK1 [239,240]. The resulting action leads to the formation of phagophores to encapsulate the mitochondria by the autophagy protein microtubule-associated protein 1 light chain 3 (MAP1LC3/LC3) [241]. The encapsulated mitochondria/phagophore formation is then matured into autophagosomes, delivering the mitochondria to lysosomes, thus removing mitochondria and ROS.

PINK1-Parkin pathway dysregulation leads to impaired mitophagy, which contributes to mitochondrial dysfunction and the progression of cardiac toxicity. Parkinson’s disease, where the Parkin name comes from, is associated with mutations in either PINK1 or PRKN [240,242]. Moreover, PINK1 is continuously imported into healthy mitochondria under normal conditions and rapidly degraded. However, when mitochondrial membrane potential is lost due to damage, PINK1 stabilises and accumulates on the OMM [243]. PINK1 is generally expressed in tissues and is undetected under basal conditions in normal cells, as determined by the mitochondrial outer membrane potential. PINK1 is typically imported via the translocase of the outer membrane (TOM) and processed by the mitochondrial processing proteinase (MPP) [244].

In diabetes, however, oxidative stress and mitochondrial dysfunction disrupt mitophagy, meaning that damaged mitochondria accumulate in cells. The failure to clear dysfunctional mitochondria results in a vicious cycle of increased oxidative stress and further mitochondrial injury. Moreover, disruptions to ATP availability and energy production, such as effects in T2DM and CVD, can contribute to a reduction in the removal of dysfunctional mitochondria as mitophagy itself leads to a reduction in mitochondrial quantity, thereby triggering the biogenesis of mitochondria in response to increased AMP/ATP and NAD^+^/NADH ratios, resulting in increased consumption of ATP [245].

In contrast, the overexpression of PINK1 attenuates cardiac myocyte apoptosis induced by hypoxia-reoxygenation, whereas the overexpression of mitophagy is known to increase apoptosis of cardiomyocytes and MI. Studies involving ischemia-reperfusion-induced myocardial cell injury have further highlighted that the overexpression of PINK1 and PARKN were able to stabilise the ETC, increase ATP production and inhibit ROS-generating mitochondria [246,247].

In cardiomyocytes, impaired mitophagy leads to the accumulation of damaged mitochondria that continue to generate ROS, disrupting cardiomyocytes’ structural and functional integrity and leading to cardiac fibrosis and hypertrophy.

### 4.4. Involvement of AMPK and mTOR Signalling

Mitochondria require a high demand for ATP, and alterations in ATP availability can be detrimental to mitochondrial functionality. AMPK and the mammalian transporter of rapamycin (mTOR) are critical regulators of the cellular energy balance, maintaining cellular homeostasis and responding to metabolic stress. An increase in AMP activates the AMPK/ATP ratio when cellular energy is depleted during pathological events, including metabolic poisoning, oxidative stress, hypoxia and nutrient deprivation to conserve ATP [248]. Once activated, AMPK promotes catabolic processes and inhibits anabolic pathways [249]. In contrast, mTOR is activated by nutrients and growth factors and upregulates cell growth, promoting protein synthesis, autophagy and cell proliferation [223]. During T2DM and CVD, ATP consumption exceeds the production, increasing adenosine diphosphate (ADP) by converting two ADPs to AMP and ATP, increasing the cytosolic concentration of AMP needed for AMPKγ subunit binding [250].

Within the functioning mitochondria, AMPK regulates cellular energy balance through α, β and γ subunits [248,249]. Human α2 and β2 subunits are expressed within the heart and skeletal muscles and are key mediators for AMPK functions [251]. AMPK activation depends on phosphorylation at Thr172 of the α subunit, while the β subunit binds myristylation, phosphorylation and glycogen [252]. AMPK regulates cellular energy homeostasis by regulating AMP through the phosphorylation of upstream calmodulin-dependent protein kinase kinase (CAMKK) and liver kinase B1 (LKB1) [251].

LKB1-AMPK signalling modulates gluconeogenic genes by regulating transcription coactivator cAMP response element-binding protein (CREB)-regulated transcription coactivator 2 (CRTC2) [253], which enhances gluconeogenic genes such as PGC1α [254]. Meanwhile, within a high-insulin state such as T2DM, insulin inhibits glucose production by suppressing cellular CRTC2 and forkhead box-O1 (FOCO1) proteins [255].

To counter this, common anti-T2DM medications such as metformin indirectly activate AMPK by inhibiting mitochondrial ETC complex I [256,257], impairing mitochondrial NADH oxidation, reducing the proton gradient and ATP synthesis and elevating mitochondrial AMP levels [258]. The increased mitochondrial AMP levels inhibit adenylate cyclase, preventing the conversion of ATP to cAMP and reducing cAMP levels, which reduces protein kinase A (PKA) activity and inhibits glucagon-dependent gluconeogenesis [259].

AMPK suppresses energy-intensive processes like protein translation and lipid biosynthesis via the inhibition of mTOR, eukaryotic elongation factor-2 (EEF2) and acetyl-CoA carboxylase (ACC). The inhibition of ACC lowers malonyl-CoA levels and thereby inhibits carnitine palmitoyltransferase-1, which is responsible for controlling the entry of long-chain fatty acid molecules into the mitochondria for oxidation [42,260].

AMPK phosphorylates the tuberous sclerosis complex (TSC-2), thereby increasing its ability to inhibit the mTOR pathway and curb cell growth during glucose starvation. The mTOR pathway is activated by growth factors and amino acids, stimulating translation by activating ribosomal protein S6 kinase (S6K1) and the phosphorylation of elongation factor-4E binding protein 1 (4E-BP1) [261]. Furthermore, AMPK activation reduces mPTP opening during reperfusion, protecting against oxidative and Ca^2+^ stress via AMPK-adenosine receptor stimulation to promote Akt activation, contributing to myocardial infarction salvage pathways [32,262].

AMPK also influences mitochondrial targets like PPARα and PGC-1α, contributing to the energy metabolism coordination within the heart since PPARs are nuclear hormone receptors that act as central cardiac fatty acid metabolism regulators [106]. PPARα, highly expressed in the heart, can directly reduce myocardial ischaemia injury and prevent mPTP formation by interacting with mPTP components, VDAC, adenine nucleotide translocase (ANT) and/or cyclophilin D (CyPD), and also indirectly by activating signalling pathways PI3K/Akt and NO production [263,264,265]. However, the inhibition or dysregulation of AMPK, such as that demonstrated during T2DM and CVDs, promotes the release of EEF2, mTOR and ACC from inhibition, increasing ATP depletion and adding to mitochondrial dysfunction [266].

As highlighted, AMPK is a critical regulator of mTOR and the mTOR complex 1 (mTORC1) [59]. The inhibition of mTOR leads to feedback activation of Akt, thereby regulating apoptosis, proliferation and cell growth [267,268]. AMPK inhibits mTORC1 by phosphorylating TSC2 and Raptor when activated during energy-deficient states and increased AMP levels [269,270]. mTORC1 inhibition activates autophagy via the initiation of the Unc-51-like autophagy activating kinase (ULK-1), which promotes the formation of the autophagy-initiating complex [45]. Hexokinase-II, the glycolytic enzyme, also binds to mTORC1, decreasing its activity during glucose deprivation [271].

mTORC1 also plays a role in sensing ATP levels and ROS signals involved in regulating cell growth and proliferation [272,273].

Enhanced mTORC1 signalling stimulates adipogenesis and the increased expression of key adipogenesis transcription factors PPARγ and SREBP1. mTORC1 is a multi-domain protein consisting of the serine/threonine protein kinase domain at the C-terminus related to PI3K; therefore, chronic mTORC1 activation can dampen the function of IRS-1, worsening insulin resistance [274]. Therapeutically, the attenuation of mTORC1 overexpression signalling aids in the prevention of the onset of T2DM and CVD [272]. From this, the dysregulation of elements of the mTORC1 pathway has been reported in various diseases and involves PI3K amplification/mutation, dysfunctional PTEN or the overexpression of Akt, placing an overwhelming burden on the SR [191]. This overload leads to SR stress and activation of the unfolded protein response (UPR), a cellular response to relieve the protein misfolding and restore SR function.

The chronic activation of mTOR and suppression of AMPK creates an environment of excessive protein synthesis, lipid accumulation and impaired autophagy. These negative effects lead to the dysregulation of RNA-binding proteins (RBPs), affecting alternative splicing, mRNA stability and translation efficiency. We can gain greater insight into therapeutic targets for diabetic heart disease by exploring the connection between RBP dysregulation and mitochondrial dysfunction.

## 5. Emerging Molecular Insights in Diabetic Cardiomyopathy

### 5.1. RNA Binding Proteins

RBPs are key regulators of RNA and play many roles in controlling post-transcriptional events in the cell [275]. They are extremely versatile proteins that regulate RNA, including splicing, localisation, translation and stabilisation [276]. RBPs also regulate the function of non-coding RNAs, therefore imposing post-transcription control. Many different chronic diseases, including diabetes, cardiovascular disease, cancer and neurodegenerative diseases, can result from RBP’s dysfunction [277].

It has been shown that RBPs can respond to different environmental signals, injuries and risk factors, including diabetes, to prevent damage, maintain homeostasis and restore function. However, the response of RBPs can also lead to the onset and perseverance of several diseases. The ability of RBPs to differentiate between health and disease states has appeared as an essential regulator of the transcriptome between cardiovascular health and disease [278].

RBPs are associated with many different diabetes complications, including diabetic neuropathy, diabetic nephropathy, diabetic cardiomyopathy, diabetic cardiovascular disease and diabetic retinopathy. More than 1200 RBPs have been verified in the human genome, along with numerous newly discovered RBPs [279]. Evidence suggests that several RBPs are involved in the regulation of mitochondrial RNA metabolism, translation and turnover [280,281,282]. Their dysregulation may contribute to the mitochondrial dysfunction observed in diabetic cardiomyopathy. Unfortunately, therapeutic approaches are limited by the current knowledge of RBPs and their role in the development of cardiovascular disease in diabetes [283]. Numerous different RBPs are implicated in diabetes, including TAR DNA-binding protein, ELAVL1, LIN28a and RBFOX2.

TAR DNA-binding protein 43 (TDP-43) is a versatile RNA/DNA-binding protein involved in RNA metabolism, playing a role in stabilising mitochondrial transcripts. When TDP-43 becomes oxidised at its cysteine RNA-binding domain, it misfolds and aggregates [284,285]. The accumulation of dysfunctional TDP-43 in diabetic conditions is associated with decreased mitochondrial gene expression and impaired ATP production [286]. In addition, RNA oxidation that alters TDP-43′s RNA-binding affinity may disrupt its nuclear functions. This translocates TDP-43 to the cytoplasm where it forms aggregates, sequestering other RNA-binding proteins and aggravating cellular dysfunction [287,288].

Another RBP associated with endothelial cell function is the embryonic lethal-abnormal vision-like protein 1 (ELAVL1 or HuR), a member of the Hu family, which regulates gene expression by stabilising mRNAs [289]. This RBP stabilises transcripts involved in oxidative stress responses, including the expression of vascular endothelial growth factor (VEGF) and hypoxia-inducible factor one alpha (HIF1α), linked to angiogenesis, apoptosis and inflammation [290]. The chronic upregulation of ELAVL1 by the PKCβ/ELAVL1/VEGF pathway is associated with the development of diabetic complications [282,291]. Under ER stress, ELAVL1 translocates from the nucleus to the cytoplasm, improving the stability of UPR-related mRNAs such as ATF4 and GRP78 and prolonging protective ER stress signalling [292]. However, chronic ELAVL1 activation in diabetes sustains pro-apoptotic UPR pathways, contributing to cardiomyocyte loss.

Another affected element is Death-associated protein 6 (Daxx), a crucial scaffold in translocating GLUT4 vesicles, boosting glucose uptake in cardiomyocytes, in addition to its role in apoptosis or cell survival, depending on its interactions [293]. Disrupting Daxx function impairs the GLUT4 vesicle delivery system and significantly reduces GLUT4 membrane translocation [294]. In one study, EVs from cultured diabetic endothelial cells were absorbed by cardiomyocytes, boosting the protein levels of mammalian sterile 20-like kinase 1 (Mst1), which plays a role in the Hippo pathway that regulates apoptosis and autophagy [295]. Mst1 prevented glucose uptake by binding to Daxx, interfering with GLUT4 membrane translocation, and directed Daxx toward apoptotic signalling.

RBPs also play an important role in the biogenesis and cargo loading of extracellular vesicles (EVs) [296]. In cardiac myopathy, dysregulated RBPs can alter the RNA and protein composition of EVs and impact their ability to mediate intercellular communication [297]. These EVs, which can be enriched with specific RNAs and RBPs, may propagate mitochondrial dysfunction and inflammation in cardiac cells if they are on the recipient side.

### 5.2. Extracellular Vesicles

EVs are nano-to-microsized lipid-bound vehicles mostly found in the form of exosomes, microvesicles and apoptotic bodies. Depending on the EV type, each specialises in size, cell surface markers, biogenesis and therapeutic potential [298]. The systematic vascular networks, rich in endothelial cells (ECs), vascular smooth muscle cells (VSMCs) and pericytes, drive the production of EV populations, which deliver lipids, nucleic acids, proteins, mRNA and miRNA as paracrine cargo [299]. Specifically, EV cargo is associated with the plasma membrane and cytosol and is involved in lipid metabolism [300]. They play a crucial role in the progression of diabetic cardiac dysfunction by mediating pathological processes such as oxidative stress, inflammation, mitochondrial dysfunction, cardiomyocyte apoptosis and fibrosis.

Diabetic macroangiopathy doubles the risk of vascular disease, including atherosclerosis and cardiovascular disease, which are known to be regulated by microRNAs. EVs, specifically exosomes, can carry between 100 and 120 different types of microRNA, and the processing and packaging of microRNA into EVs is highly controlled and minimises extracellular waste [301].

EVs contribute to cardiomyocyte apoptosis by transferring pathogenic microRNAs (miRNAs) that regulate cell survival pathways. miR-130b-3p, which is highly expressed in diabetic adipocyte-derived EVs, is able to bind to the 3′ untranslated region (3′UTR) of *PGC-1α* mRNA, leading to its degradation or translational repression. Suppressing *PGC-1α* expression leads to mitochondrial dysfunction and increased ROS generation [302].

As mentioned earlier, exosomal Mst1 from endothelial cells promotes apoptosis by inhibiting autophagy and insulin signalling, thereby impairing GLUT4 membrane translocation and insulin resistance [295].

Studies conducted both in vitro and in vivo have demonstrated that miR-25 levels increase in heart failure, playing a crucial role in inhibiting SERCA2a and disrupting Ca^2+^ uptake during heart contraction. Consequently, it hinders intracellular Ca^2+^ management in cardiomyocytes by slowing the calcium uptake that is dependent on SERCA2a [303].

In atherosclerosis, microRNA-221/222 is responsible for the pro-inflammatory differentiation of monocytes to M1 macrophages, which contributes to plaque development in the arteries, resulting in myocardial infarction and stroke [304].

These cargoes may enhance the biological processes of recipient ECs, including reducing inflammation, apoptosis, migration, angiogenesis and proliferation, thus maintaining endothelial homeostasis. Recently, targeting microRNAs for therapeutic impact has been brought to the forefront.

Metformin is typically used to treat T2DM and directly minimises microRNA-221/222. Thus, it reduces the production of foam cells and prevents diabetic-mediated cardiovascular complications [304]. In T2DM, microRNA-144-3p suppresses NRF2, a redox regulatory gene that protects cells from oxidative stress. However, upon NRF2 suppression by microRNA-144-3p, insulin resistance is exacerbated. However, when treated with the circRNA, a form of the exosome circ-Snhg, insulin resistance is nullified, and wound healing is promoted [305].

The previously discussed mechanisms underlying diabetic cardiomyopathy highlight the complexity of this disease. Traditional cell culture models and animal studies are not able to fully recapitulate human-specific disease processes due to the multifactorial nature of diabetes-induced cardiac toxicity. This is where novel human models, such as patient-specific iPSC-derived organoids, emerge as transformative tools.

## 6. Therapeutic Strategies and Future Directions

### 6.1. iPSC-Derived Cardiac Organoids

Diabetes and diabetic-associated cardiac complications are a complex and vital area that requires attention and innovative techniques to enhance our understanding and improve pharmacological intervention.

**Figure 9:**
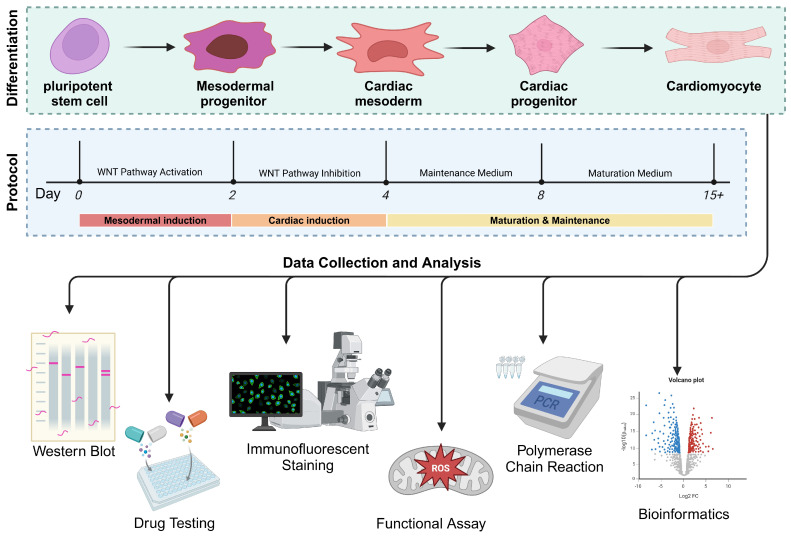
iPS-derived cardiac differentiation protocol and application. The differentiation process of pluripotent stem cells into cardiomyocytes begins as they are directed through various stages: mesodermal progenitor, cardiac mesoderm, cardiac progenitor and, finally, cardiomyocytes. The matured cardiac organoids can undergo experiments for data collection and analysis. Lab techniques include Western blot, drug testing, immunofluorescent staining, ROS measurement, polymerase chain reaction (PCR) and bioinformatics tools. Created with BioRender.com.

Significant distinctions exist when comparing cardiomyocytes from small animal models and human cardiomyocytes, including beating rates, Ca^2+^ handling, myofilament composition and cellular electrophysiology. These physiological differences are substantially less between humans and large animal models such as primates, pigs and dogs [306]. The recent improvement in culturing iPSCs technology with the ability to direct their differentiation into specific cell types, such as cardiomyocytes, presents a remarkable advantage in diabetic research for modelling patient-specific cells that can be used in drug screening and regenerative medicine, as summarised in Figure 9 [307,308].

Multiple protocols have successfully developed iPS-COs to replicate the structure and function of the heart. They have been engineered to incorporate essential myocardial components that enhance their physiological relevance, which include cardiac progenitor cells, fibroblasts, endothelial cells and neuronal elements. Developing iPS-COs to resemble the human heart and heart components closely has allowed them to be used in several research areas, allowing for detailed investigations of heart diseases like cardiomyopathy, arrhythmias and ischemic heart disease. This approach allows for a deeper exploration of epigenetic modifications in molecular signalling, which can be targeted using CRISPR-Cas9 or pharmacological interventions prior to in vivo studies.

Various types of cardiac organoids have been developed in the literature, each with distinct characteristics. The following list mentions the latest models:

1. Co-cultured COs: Engineered to include neuronal cells or endothelial networks, modelling neuro-cardiac interactions or mimicking in vivo vascularisation [309].
2. Self-assembled chambered COs [310,311]: These CCOs exhibit stable chambers with self-organised outer myocardial and inner endocardial layers encapsulating a central cavity. Functionally, they can recapitulate clinical cardiac hypertrophy by exhibiting thickened chamber walls, reduced fractional shortening and increased myofibrillar disarray.
3. Multi-chambered self-paced vascularised COs [312]: A mixture of iPSC-CMs and rat primary cardiac microvascular endothelial cells CECs was seeded in geometrically confining microwells. The anisotropic stress gradient distribution in vascularised organoids results in a multi-chambered cardiac organoid formation.
4. 3D-bioprinted chambered COs [313]: Similar to aggregate-based organoids, but with the key benefit of containing geometric structures crucial for cardiac muscle pump function since they are printed as an anatomical heart shape.

Prior research has suggested that in order to model diabetic cardiomyopathy using human iPS-COs, the cells should exhibit the essential characteristics associated with the development of this disease condition, including insulin resistance, a metabolic shift, lipotoxicity and cardiac dysfunction alterations [20]. Studies have used several techniques to drive these cardiomyocytes into stressful conditions resembling diabetic dysfunction, such as using high-glucose or high-fat media [20,314].

However, as our team previously reported, iPSCs derived from diabetic patients have shown epigenetic memory [315] able to influence the cellular functions to develop the same genetic and metabolic dysregulations occurring in the original donor as they differentiate into mature cells [316,317]. These epigenetic alterations allow the iPSCs to be cultured in non-diabetic conditions with minimal stressful conditions, and as iPS-Cos, they would still exhibit a full spectrum of altered functions similar to the diabetic picture under current investigation [318,319].

Differentiated cardiomyocytes derived from healthy and diabetic donors can be used as a disease model that holds great potential for therapeutic target exploration within the diabetic population. Experimental therapies can be administered at a large scale or tailored to individual patients based on their unique genetic and epigenetic profiles.

### 6.2. Future Directions

Future studies on diabetes cardiomyopathy should focus on employing advanced iPS-COs models to examine disrupted signalling pathways, specifically because they offer a platform that mimics disease characteristics such as mitochondrial dysfunction, lipotoxicity and disrupted Ca^2+^ management. Moreover, they allow for the investigation of how RNA-binding proteins regulate RNA stability and translation, which are vital for addressing the cellular dysfunction involved in this diabetes condition.

Different strategies can target RBPs, including RNA–protein interactions, protein–protein interactions, protein aggregation and cell pathways [320]. The current strategies involve either manipulating a specific RBP, considered a direct method, or a specific RBP–RNA interaction, considered an indirect method. Direct methods involve either the overexpression or knockdown of a particular RBP [282]. Indirect methods involve the use of circular RNA, siRNA, synthetic peptides, oligonucleotide-based aptamers, small molecules and CRISPR, which can be designed to either inhibit the RBP’s ability to interact with RNA by inducing degradation, suppressing enzymatic activity and preventing post-transcriptional modification or bind to outcompete a chosen RBP.

Regarding RBP therapies, many are currently in lab and clinical assessment, although they are still in the early stages of development. Using RBPs as therapies may provide a better strategy to current methods, as exploiting RBPs could help repair damage and regenerate any affected cells, such as endothelial cells, caused by diabetic cardiovascular complications. The advantage of targeting RBPs is their specific binding structure, which allows for a particular RBP to be targeted, reducing the risk of off-target effects. While these RBPs were studied across different physiological states, those linked to oxidative stress also led to posttranscriptional modifications and played vital roles in cardiac cells [321].

Research on EVs also holds great potential because they enable intercellular communication and transport molecular cargo that mirrors the diabetic environment. EVs derived from diabetic sources might show changes in RNA and protein content driven by RBP. This could lead to mitochondrial dysfunction and disrupted signalling to recipient cells, including endothelial cells and fibroblasts. Enhanced CO models combined with high-throughput omics technologies may uncover the role of EV cargo composition in disease progression.

These models could be enhanced with vascularisation and microfluidics to offer a more accurate representation of the diabetic microenvironment in the human body and to help us understand the impacts of hyperglycaemia and hyperlipidaemia on heart disease. CRISPR-Cas9 gene-editing techniques can be implemented to focus on epigenetic changes and RBP-related disruptions, which could elucidate the mechanisms behind diabetic cardiomyopathy. These improvements will support the creation of tailored, patient-specific treatments targeting the core mechanisms of the disease.

## Data Availability

Not applicable.

## Abbreviations

3’UTR	3’ Untranslated Region
ADP	Adenosine Diphosphate
AGE	Advanced glycosylated end-products
AIF	Apoptosis-Inducing Factor
AKT	Protein Kinase B
ALDH2	Aldehyde Dehydrogenase 2
AMPK	AMP-activated Protein Kinase
ANP	Atrial Natriuretic Peptide
ANT	Adenine Nucleotide Translocator
ATP	Adenosine Triphosphate
BAX	BCL2-associated X protein
Bcl-2	B-cell lymphoma 2
CAMKK	Ca^2+^/Calmodulin-dependent Protein Kinase Kinase
CAT	Catalase
CD36	Cluster of Differentiation 36
CHOP	C/EBP Homologous Protein
COPD	Chronic Obstructive Pulmonary Disease
CRTC2	CREB-Regulated Transcription Coactivator 2
CVD	Cardiovascular Disease
DAG	Diacylglycerol
DRP1	Dynamin-related protein 1
ER	Endoplasmic Reticulum
ETC	Electron Transport Chain
FA	Fatty Acid
FAO	Fatty Acid Oxidation
FIS1	Mitochondrial fission protein 1
FOCO1	Forkhead Box O1
GAP	Glyceraldehyde-3-phosphate
GAPDH	Glyceraldehyde-3-phosphate Dehydrogenase
GLUT1	Glucose transporter type 1
GLUT4	Glucose Transporter Type 4
GRP78	Glucose-regulated Protein 78
GlcNAc	N-acetylglucosamine
HF	Heart Failure
H^+^	Proton
ICAD	Inhibitor of Caspase-Activated DNase
IRE1	Inositol-requiring Enzyme 1
JNK	c-Jun N-terminal Kinase
LKB1	Liver Kinase B1
LTCC	L-type Calcium Channel
MAPK	Mitogen-activated Protein Kinase
MCU	Mitochondrial Ca^2+^ uniporter
MFF	Mitochondrial fission factor
MFN1	Mitofusin 1
MFN2	Mitofusin 2
MI	Myocardial Infarction
MPP	Mitochondrial Processing Peptidase
MiD49	Mitochondrial dynamics proteins 49
MiD51	Mitochondrial dynamics proteins 51
NCX	Sodium-Calcium Exchanger
NOX	NADPH Oxidase
NRF1	Nuclear respiratory factors 1
NRF2	Nuclear Factor Erythroid 2-Related Factor 2
OGT	O-GlcNAc Transferase
OPA1	Optic atrophy 1
ORAIP	Oxidative Stress-Responsive Apoptosis Inducing Protein
PARP-1	Poly(ADP-Ribose) Polymerase 1
PGC-1α	Peroxisome proliferator-activated receptor gamma coactivator 1-alpha
PINK1	PTEN-induced putative kinase 1
PIP2	Phosphatidylinositol 4,5-Bisphosphate
PKA	Protein Kinase A
PKC	Protein Kinase C
PPARs	Peroxisome proliferator-activated receptors
PTEN	Phosphatase and Tensin Homolog
RAGE	AGE receptors
RBP	RNA-binding Protein
ROS	Reactive Oxygen Species
RyR2	Type-two ryanodine receptor
S6K1	Ribosomal Protein S6 Kinase 1
SERCA	Sarco/Endoplasmic Reticulum Calcium-ATPase
SGLT1	Sodium-Glucose Cotransporter 1
SOICR	Store Overload-Induced Ca^2+^ Release
SR	Sarcoplasmic reticulum
T2DM	Type 2 Diabetes Mellitus
TDP-43	TAR DNA-binding Protein 43
TGF-β	Transforming Growth Factor Beta
TOM	Translocase of the Outer Membrane
UCP	Uncoupling Protein
ULK-1	Unc-51 Like Autophagy Activating Kinase 1
UPR	Unfolded Protein Response
VDAC	Voltage-Dependent Anion Channel
VEGF	Vascular Endothelial Growth Factor
cAMP	Cyclic Adenosine Monophosphate
iPSC	Induced Pluripotent Stem Cells
mPTP	Mitochondrial permeability transition pores
